# Mitochondria: a key regulator of programmed cell death in OP

**DOI:** 10.3389/fendo.2025.1576597

**Published:** 2025-07-02

**Authors:** Kexin Wang, Zhandong Wang, Chunhua Ma, Junfang Yang, Shangman Xing, Bing Song, Chao Guo, Wenjing Song, Tingting Cao, Min Bai, Yongfeng Wang

**Affiliations:** ^1^ The First Clinical Medical College, Gansu University of Chinese Medicine, Lanzhou, China; ^2^ Clinical College of Integrated Traditional Chinese and Western Medicine, Gansu University of Chinese Medicine, Lanzhou, China; ^3^ Medicine Research and Experimental Center, Gansu University of Chinese Medicine, Lanzhou, China; ^4^ College of Traditional Chinese Medicine, Ningxia Medical University, Yinchuan, China; ^5^ School of Basic Medicine, Gansu Medical College, Pingliang, China

**Keywords:** osteoporosis, mitochondria, programmed cell death, apoptosis, necroptosis, pyroptosis, ferroptosis, mitophagy

## Abstract

Osteoporosis (OP) is a common clinical systemic bone disease, with insidious onset and usually causes serious complications such as fractures. Studies have found that the dysfunction of a variety of bone cells will lead to enhanced bone resorption and reduced bone formation capacity, thus resulting in the imbalance of bone homeostasis and OP disease. As a class of regulatory death mode that affects cell function, programmed cell death (PCD) has been proved to play an important role in maintaining various bone cells growth activities and maintaining bone homeostasis. In addition, several studies have shown that mitochondria are important regulators of a variety of PCD, and various drugs can target mitochondria to regulate the programmed death of bone cells, which is of great significance to further explore the pathogenesis of OP and look for new and efficient drugs for OP.

## 1 Introduction

Osteoporosis (OP) is a common systemic disease of the skeletal system, and is usually considered to be associated with bone metabolic disorders in patients, mainly reflected in the imbalance between bone resorption and bone formation. A global epidemiological study of spinal fractures found that about 200 million people worldwide are affected by OP (1). The clinical manifestations of OP include the change of bone microstructure, reduced bone mass, decreased bone strength, and increased bone fragility, which can increase the risk of fracture. However, the development of OP is hidden, and most patients have developed some irreversible injuries such as mobility disorders, which will also bring serious burden to their families and society. Nowadays, with the growth of population and the development of economy, the social demand for labor is increasing, and the demand for OP treatment is becoming more and more urgent. At present, there are many clinical drugs for OP, but these drugs are expensive or have serious side effects. There is few safe and efficient drug to improve the development of OP. Therefore, it is of great significance to study and summarize the potential mechanisms of the development of OP, to explore the targets of the key mechanisms of OP, and to find new and efficient drugs for the treatment of OP.

Various types of PCD pathways play an important role in OP, which affect the development of OP by regulating the growth activity of different types of bone-related cells, and then regulating bone homeostasis. In the traditional sense, cell death can be divided into two main types, accidental cell death (ACD) and regulated cell death(RCD) (2). ACD is a biologically uncontrolled process^[3]^, generally caused by severe physical, chemical, and mechanical stimulation of cells, but ACD is difficult to prevent or regulate, so it cannot be used as a direct target for the treatment and intervention of the disease (3). RCD, distinguished from ACD, has a precise signaling cascade regulated by specific positive-negative or bidirectional effector molecules, and also has unique morphological features, biochemical features, functional and immunological consequences (4). And can use drugs or genetics to alter its course by intervening for critical parts of a specific mechanism in the RCD. Generally, the RCD occurring in a fully physiological context is called “PCD” (3). PCD has been widely studied as an adjustable and actively orderly mode of death of cells. From morphological, biochemical and functional perspectives, common PCD includes apoptosis, necroptosis, pyroptosis, ferroptosis and autophagy (5). Genes determine their ability to actively participate in maintaining normal cellular metabolism and *in vivo* environmental homeostasis. The use of these PCD pathways has significant flexibility, and their molecular regulation is quite plastic, each with special morphology, regulatory molecules, cellular processes, and outcomes; but different types of PCD can also be regarded as a single and coordinated cell death system, and can flexibly compensate each other to affect cell growth (6). Various PCD pathways in various bone tissue-related cells (bone marrow mesenchymal stem cells (BMSCs), osteoblasts, osteoclasts, osteocyte, bone marrow-derived macrophages (BMDMs), etc. are closely associated with the disease progression of OP. The PCD system of various cells related to OP is of great significance for promoting the research of new therapies for the prevention and treatment of OP, so as to effectively affect the process of OP.

Multiple evidence suggests that mitochondria is an important regulator of a variety of programmed cell death. As one of the important cytoplasmic organelles, mitochondria has its own unique features as a means to maintain its health in response to different signals, such as bilayers, unique genetic material, ATP production through oxidative phosphorylation, and the presence of multiple quality control checkpoints (7) (8). Mitochondria, also known as the power source of cells in all eukaryotes, can not only create energy for cells, but also because their special structure plays an important role in regulating cellular biological processes, including cell metabolism and apoptosis (9). Extensive evidence from preclinical and clinical studies suggests that mitochondria can affect the development of OP by participating in regulating programmed cell death, so targeting mitochondria to regulate cell death shows great potential in the treatment of OP (10). Based on the key role of mitochondria in various cell death, we discuss the mechanisms by which mitochondria regulate multiple programmed cell death patterns mediating the development of OP diseases, providing a new direction for the pathological mechanism of OP and providing more effective strategies for the clinical treatment of OP.

## 2 Apoptosis and OP

### 2.1 Definition of apoptosis

Apoptosis, also known as type I PCD, is one of the most common mechanisms of cell death (7). In the early stages of apoptosis, cell shrinkage can be observed by light microscopy (8). As cells contract, they are smaller, with dense cytoplasm, organelles accumulate more tightly, and chromatin condensation occurs (7). Subsequently, the cell plasma membrane blebs, the nucleus breaks and tightly surrounds the cell debris, and finally the surrounding cells undergo phagocytic clearance of the apoptotic bodies of the dying cells. Apoptosis usually occurs during body development and aging, mainly to maintain the homeostasis of the cell population in the tissue, and it also occurs as a defense mechanism in situations such as immune responses or when cells are damaged by toxic substances (11). The occurrence of apoptosis is mainly dependent on the sequential activation of a series of cysteine-aspartic proteases: the central regulator of apoptosis caspases will be activated from the inactive state under appropriate conditions, by autocatalytic lysis to activate itself or lysis and activate other caspases, then start the downstream proteases, thus splitting many cellular proteins, produce irreversible apoptosis, finally lead to cell PCD (12). Due to the different initiation phase, apoptosis is divided into three main regulatory pathways: intrinsic pathway, extrinsic pathway, and perforin/granzyme pathway (13). Among them, the extrinsic pathway can cross-respond with the intrinsic pathway through caspase 8 mediated hydrolysis and cleavage of the BID protein (Bcl-2 homology 3 interacting domain death agonist, BID) (14).

### 2.2 Mitochondria as the central hub for apoptotic regulation

**Figure 1:**
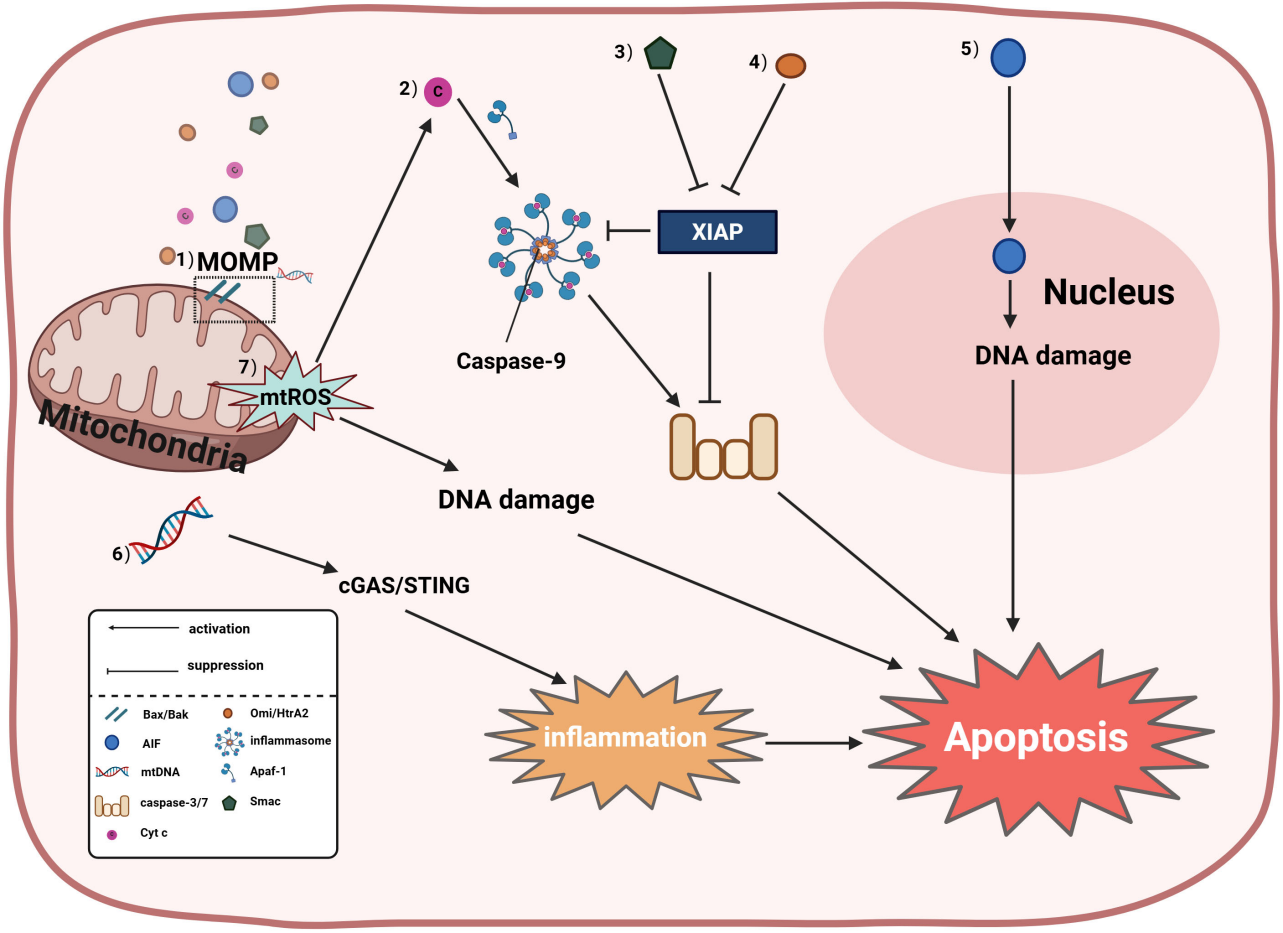
Mitochondria induce apoptosis through both caspase-dependent and caspase- independent pathways; specifically: (1) Mitochondrial outer membrane permeabilization (MOMP) is dynamically regulated by Bcl-2 family proteins, wherein activation of pro-apoptotic proteins Bax/Bak induces MOMP to release cytochrome c (Cyt c), second mitochondria- derived activator of caspases (Smac), mitochondrial DNA (mtDNA), and apoptosis-inducing factor (AIF); (2) Cyt c-dependent apoptosis involves apoptosome formation by Cyt c with apoptotic protease-activating factor 1 (Apaf-1), activating the caspase cascade (caspase-9 and caspases-3/7); (3) Smac antagonizes X-linked inhibitor of apoptosis protein (XIAP) to relieve caspase inhibition; (4) High temperature requirement factor A2(Omi/HtrA2) can promote apoptosis by disrupting XIAP-mediated caspase suppression; (5) AIF directly translocates to the nucleus to trigger DNA fragmentation independent of caspase activity; (6) When caspases are inactivated, leaked mtDNA activates the cyclic GMP-AMP synthase (cGAS)/stimulator of interferon genes (STING) pathway to drive inflammation-associated apoptosis; (7) Mitochondrial reactive oxygen species (mtROS) exert dual pro-apoptotic effects: accumulated mtROS promotes Cyt c release to activate caspase-dependent apoptosis, while also inducing DNA damage-dependent apoptosis via oxidative DNA damage and subsequent activation of the ataxia telangiectasia mutated-checkpoint kinase 2-tumor protein p53 (ATM-CHK2-p53) pathway.

Mitochondria serve as the central hub for apoptosis regulation, inducing cell death through both caspase-dependent and caspase-independent pathways (**Figure 1**). Mitochondria primarily mediate apoptosis via the intrinsic mitochondrial pathway, which is triggered by cellular stress or developmental stimuli and regulated byB-cell lymphoma-2 (Bcl-2) family proteins; all members of this family contain at least one Bcl-2 homology (BH) domain and determine cellular fate by controlling mitochondrial outer membrane permeabilization (MOMP). The Bcl-2 protein family includes pro-apoptotic members (Bax, Bak, Bok, Bim, Bik, Bad, Noxa, and Puma) and anti-apoptotic members (Bcl-2, Bcl-XL, and Mcl-1) (10, 15). Activation of pro-apoptotic proteins Bax and Bak induces pore formation in the outer mitochondrial membrane, leading to MOMP; upon MOMP occurrence, cytochrome c (Cyt c) is released into the cytoplasm, where it acts as a key factor in the intrinsic apoptotic pathway by binding apoptotic protease activating factor 1 (Apaf-1) to activate caspase-9 and form the apoptosome, subsequently activating caspases-3/7 to execute apoptosis (16, 17). Additionally, other mitochondrial factors induce apoptosis: second mitochondria-derived activator of caspases (Smac/DIABLO), apoptosis-inducing factor (AIF), mitochondrial DNA (mtDNA), and mitochondrial reactive oxygen species (mtROS). Smac/DIABLO, designated as “second mitochondria-derived activator of caspases,” interacts with the X-linked inhibitor of apoptosis protein (XIAP) to relieve its suppression of caspases-3/7/9, facilitating the caspase cascade, while the high temperature requirement factor A2(Omi/HtrA2)also promotes apoptosis by disrupting XIAP-mediated caspase inhibition (17). When caspases are inactivated, mtDNA leakage into the cytosol activates cGAS-STING signaling, releasing inflammatory factors and promoting inflammation-associated apoptosis (18). Furthermore, AIF functions as a caspase-independent death effector that translocates from mitochondria to the nucleus to trigger chromatin condensation and DNA fragmentation, inducing apoptosis (19). Excessive mtROS accumulation on mitochondria promotes cytochrome c release to induce caspase-dependent apoptosis (20), while mtROS buildup may also cause apoptosis through DNA damage (21, 22).

### 2.3 Mitochondria-mediated apoptosis in the pathogenesis of OP

Apoptosis is a crucial part of bone renewal, repair, and regeneration. The balance of cell proliferation, differentiation and apoptosis in the bone marrow affects the bone growth and development status, so the study of apoptosis of cells in the bone marrow is very necessary in the study of OP. Multiple studies further indicate that mitochondria participate in the apoptosis of OP-associated cells and play an important role in the progression of OP (**Supplementary Table 1-1**). First, the involvement of mitochondria in the mitochondrial apoptosis pathway in cells in the bone marrow affects OP, mainly reflected in osteoblasts and BMSCs. Zinc deficiency was shown to lead to the translocation of Bax into mitochondria in osteoblast cell lines, which leads to decreased mitochondrial membrane potential and increased mitochondrial membrane permeability, release of cytochrome C and AIF, and activation of mitochondria-associated caspase-dependent and independent pathways leading to osteoblast apoptosis. This may explain the association of reduced bone mass in OP with lower levels of skeletal zinc (23). Glucocorticoid-induced bone loss is the most common form of secondary OP, studies showed that long-term incubation of glucocorticoids has a substantial effect on osteoblast mitochondria, leading to cytochrome C release and mitochondrial breakdown, leading to osteoblast apoptosis (24), and another study confirmed that glucocorticoid-induced bone loss in secondary OP is the accumulation of mitochondrial overactivation and oxidative stress to induce osteoblast apoptosis, regulating mitochondrial function and biogenesis to improve mitochondrial apoptosis of osteoblasts is considered an ideal strategy to prevent glucocorticoid-induced OP (25). Mitochondrial apoptosis pathway also participates in iron overload in osteoblasts and BMSC, causing increased mitochondria-related apoptosis and damage cell osteogenesis and mineralization (26). In addition, mitochondria can directly regulate the apoptosis pathway of osteocytes and affect the process of OP. In clinical studies, it was found that mitochondrial DNA mutations are also associated with low bone mass (27, 28), and the downregulation of mitochondrial transmembrane protein OPA can induce mitochondrial ATP production and inhibit P38 signaling pathway to inhibit bone cell apoptosis and improve OP (29).

### 2.4 Targeting mitochondrial apoptosis: a therapeutic strategy for bone-related disorders

With the gradual clarity of the mechanism of OP disease occurrence, the reports of targeting mitochondria to regulate cell apoptosis related to OP disease to improve bone blood supply, alleviate bone loss, and promote bone formation are increasing year by year. Some commonly used clinical drugs have been proved to act on mitochondria-related apoptosis in OP., here we summarize relevant research (**Supplementary Table 1-2**). For example, the traditional hypoglycemic drug metformin (Metformin) was found to alter H_2_O_2_ to upregulate SIRT 3 expression through Phosphatidyl-inositol 3-kinase/Serine-threonine kinase (PI3K/AKT) pathway, so as to repair mitochondrial damage, and finally reverse osteoblast apoptosis (30). Estrogen can reduce osteocast numbers by weakening mitochondrial oxidative phosphorylation and ATP production of early osteocast precursors and promote mitochondrial apoptotic death of early osteoclast progenitors (31). Some Chinese medicine monomer can also improve OP by acting on mitochondrial apoptosis, such as natural flavonoids naringoside was found by blocking the endoplasmic reticulum stress and mitochondria-mediated apoptosis pathway to inhibit cell apoptosis in the vascular endothelium, thus regulating endothelial cell function, promote the apoptosis of osteoclasts, and then play a role of anti-OP disease (31, 32). Vanillin activates mitochondria-dependent cell apoptosis and suppresses bone resorption by inducing cytochrome c, lysed Caspase-3, BAX, and Apaf-1 (33). Euphorbia factor L1, isolated from Euphorbia, can inhibit ovariectomy-induced bone loss in mice by reducing the level of reactive oxygen species and regulating PGC-1 β that affects mitochondrial biogenesis, and inducing apoptosis of osteoclasts (34). Virgin olive oil of the important compound hydroxytyrosol (HT) can inhibit oxidative stress-induced mitochondrial dysfunction by reducing OPA 1 (Optic atrophy 1) cleavage and increasing AKT (protein kinase B) and GSK 3 β (Glycogen synthase kinase 3β) phosphorylation, Improve the bone cell apoptosis (35). Puerarin, sulforaphane, tanshinone IIA, curcumin, and grape seed procyanidins can also become important targets for improving OP by inhibiting mitochondria-related cell apoptosis (36–40). Silbinin can downregulate the expression of late glycoclation end product receptor and its mediated cellular mitochondrial apoptosis pathway, Thus preventing the osteoblast apoptosis induced by late glycation end products (41); crocin can prevent dexamethasone-induced osteoblast mitochondrial pathway apoptosis through the ROS/Ca^2+^ pathway, And then to improve the secondary OP (42); The notoginsenoside R1 isolated in notoginseng can restore oxidative stress-induced mitochondrial membrane potential and abnormal ROS levels by inhibiting the JNK signaling pathway, Promote the osteogenic differentiation of osteoblasts (43); selenium-containing protein from selenium-enriched spirulina platensis reduce osteoblast apoptosis by inhibiting mitochondrial dysfunction and ROS-mediated oxidative damage (44). Fermented oyster extract can regulate mitochondrial dysfunction and improve oxidative stress-induced osteoblast apoptosis through the AKT-GSK 3 β signaling pathway (45). The first-generation statin drug, simvastatin, protected human osteosarcoma cells from oxidative stress-induced apoptosis through mitochondria-mediated signaling (46), and astragalus polysaccharide, the main active ingredient of astragalus, alleviated iron overload-induced BMSC dysfunction by inhibiting mitochondrial ROS (47). In addition, other ways to regulate mitochondria are also involved in the regulation of OP-related apoptosis. For example, vesicles of proteus mirabilis Vesicles can promote mitochondria-dependent apoptosis by regulating the miR 96-5p/Abca 1 axis, thus inhibiting bone loss in osteoclastogenesis and ovariectomized mouse models (48). Inhibition of HMGB can alleviate high glucose-induced apoptosis in BMSC cells by targeting activating the AMPK pathway and relieving mitochondrial dysfunction, and related inhibitors may serve as effective agents for diabetic OP (49). The mitochondria-derived cell-protective polypeptide, HN(Humanin), encoded by mtDNA, can prevent cell apoptosis, Its analogue HNGF6A can effectively prevent osteoblast apoptosis and restore the osteogenic differentiation ability of osteoblasts (50).

## 3 Necroptosis and OP

### 3.1 Definition of necroptosis

Necroptosis, also called programmed necrosis, initially first defined by Degterev et al. (51), belongs to nonapoptotic cell death independent of the caspase pathway. Necroptosis is a process of regulated necrotizing cell death mediated by RIP1 (Receptor-Interacting Protein 1) and RIP 3 (Receptor-Interacting Protein 3) kinases, which differs from apoptosis and other forms of programmed necrotic cell death, operates independently of caspase activity and represents a self-destructive mechanism activated when apoptosis is compromised. Various stimuli can trigger necroptosis in cells, including the activation of Tumor Necrosis Factor Receptor (TNFR) superfamily members, Pattern Recognition Receptors (PRRs), and T cell receptors. These stimuli ultimately induce necroptosis by activating pathogen-associated molecular patterns (such as RIG-I-like receptors or Toll-like receptors) or responding to signals like oxidative stress. (4). In the activation pathway of necroptosis, TNF α/TNFR (Tumor necrosis factor- α/Tumor necrosis factor receptor) signaling pathway is considered one of the most deeply studied ways. Typically, extracellular stimuli trigger ligand binding and activate death receptors such as TNFR1 in the cell membrane, where the receptor recruits RIPK1 (Receptor-interacting serine/threonine-protein kinase 1) to its intracellular domain via adaptor proteins TRADD and TRAF2, followed by ubiquitination through ubiquitin ligase cIAP1/2; ubiquitinated RIPK1 activates the NF-κB (Nuclear factor kappa-B) pathway through the IKK (inhibitor of kappa B kinase) complex, thereby promoting cell survival. Upon removal of ubiquitin chains by deubiquitinating enzymes, released RIPK1 undergoes autophosphorylation and conformational changes that expose its oligomerization domain, enabling recruitment and activation of RIPK3 (Receptor-interacting serine/threonine-protein kinase 3); activated RIPK3 phosphorylates MLKL (Mixed Lineage Kinase Domain-Like), leading to MLKL oligomerization and translocation to the plasma membrane, where it induces phospholipid scrambling, disrupts membrane integrity, and forms pores, resulting in Ca²^+^/Na^+^ influx and release of cytokines and DAMPs (Damage-associated molecular patterns), ultimately causing plasma membrane rupture and cell death. Besides the TNFR1 pathway, RIPK3 is oligomerized by TRIF (Toll/IL-1 receptor domain-containing adaptor inducing IFN- β) and activated by ZBP 1 (Z-DNA-binding protein 1) to promote Necroptosis. Oligomerized RIPK3 also binds RIPK1 and then recruitment disrupts the FADD-caspase-8-c-FLIP complex, and Necroptosis occurs when the function of the caspase-8-c-FLIP dimer is inhibited or disrupted (52). Characteristic alterations in necroptosis include early loss of plasma membrane integrity, release of cellular contents, and organelle swelling. In-depth investigation into the regulatory mechanisms of this cell death modality will offer novel therapeutic perspectives for inflammatory diseases and conditions such as osteoporosis.

### 3.2 Mitochondrial multi-pathway regulation of necroptosis

**Figure 2:**
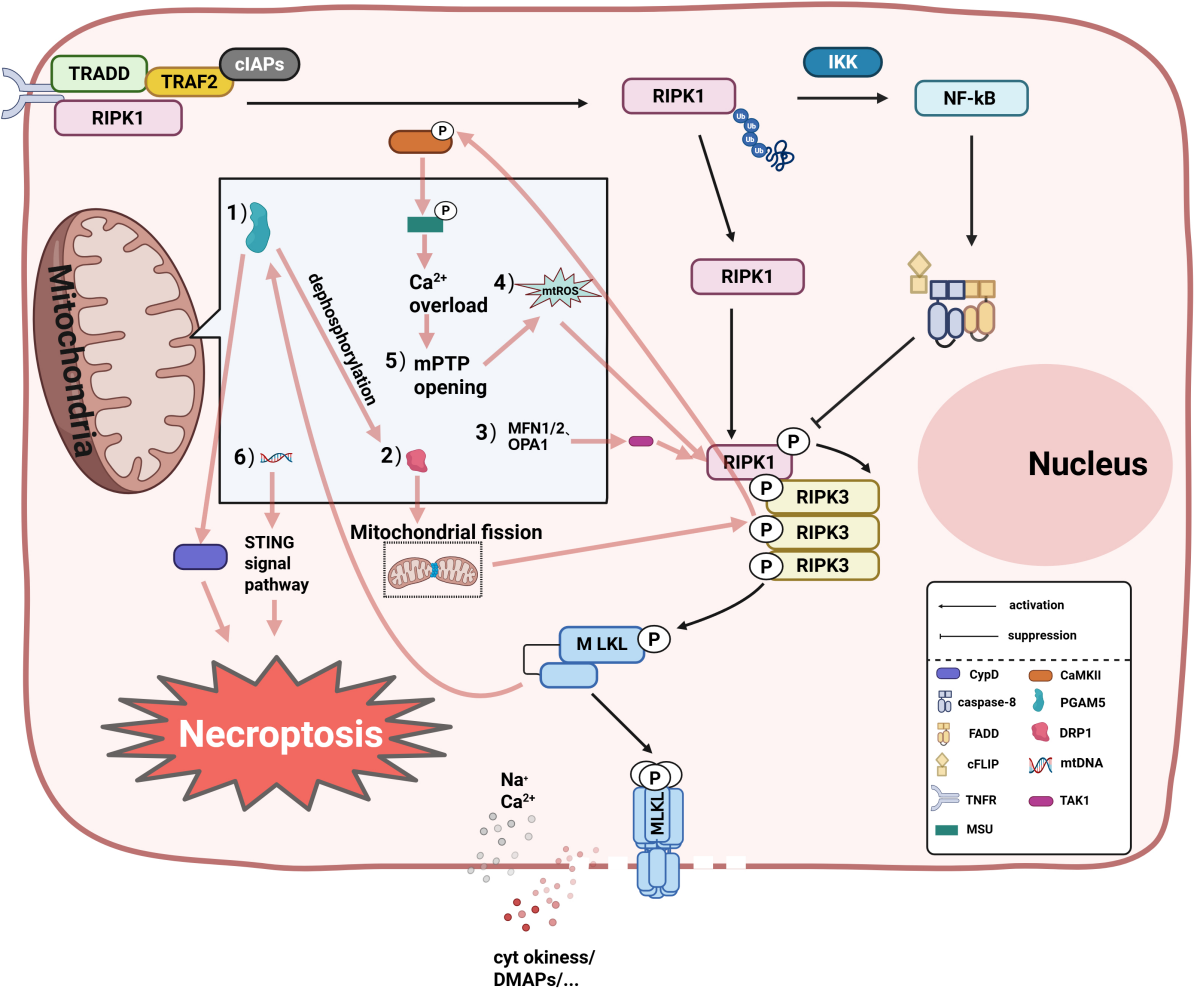
The specific mitochondrial regulatory mechanisms in necroptosis are as follows: (1) Dynamin-Related Protein 1 (DRP1)-dependent mitochondrial fission is enhanced through dephosphorylation by Phosphoglycerate Mutase Family Member 5 (PGAM5), promoting necrosome signal transduction; (2) Acting downstream of RIPK3/MLKL, PGAM5 amplifies necroptosis by activating Cyclophilin D (CypD) to facilitate mitochondrial permeability transition pore (mPTP) opening; (3) Mitofusins 1/2 (MFN1/2) and Optic Atrophy 1 (OPA1) suppress necroptosis by inhibiting TGF-β-Activated Kinase 1 (TAK1)-mediated phosphorylation of Receptor-Interacting Protein 1 (RIPK1); (4) mtROS accumulation: mtROS activates Receptor- Interacting Protein Kinase 1/3 (RIPK1/RIPK3) via oxidative modification, forming necrosome complexes that drive Mixed Lineage Kinase Domain-Like Protein (MLKL) phosphorylation and plasma membrane pore formation; (5) mPTP opening: RIPK3 activates Calcium/Calmodulin- Dependent Protein Kinase II (CaMKII), which phosphorylates the Mitochondrial Calcium Uniporter (MCU), causing mitochondrial Ca²^+^ overload that triggers mPTP opening. This process induces mtROS accumulation and necroptosis;(6) mtDNA release: MLKL-mediated membrane permeabilization facilitates mtDNA efflux, activating the STING- dependent pronecrotic signaling cascade through the TANK-Binding Kinase 1 (TBK1)-Interferon Regulatory Factor 3 (IRF3) pathway, thereby amplifying inflammatory responses. NF-κB, Nuclear factor kappa-B; IKK, Inhibitor of kappa B kinase complex; TRADD, Tumor Necrosis Factor Receptor Type 1-associated death domain protein; TRAF2, TNF receptor-associated factor 2; FADD, Fas-associated death domain protein; cIAPs, Cellular inhibitor of apoptosis proteins.

Recent studies show a direct association between mitochondria and cell Necroptosis (53). First, plasma membrane permeabilization induced by MLKL usually leads to pore formation by ion influx, releasing mtDNA (53) and other factors to affect necrotitic apoptosis of cells. Second, multiple studies indicate that the accumulation of mtROS, mitochondrial function-related proteins, opening of the mitochondrial permeability transition pore (mPTP), and release of mtDNA collectively regulate cellular necroptosis (**Figure 2**). Research by Zhang et al. found that elevated mtROS activates RIP kinases, leading to the recruitment of RIPK3 by RIPK1, forming a functional necrosome that induces necroptosis (54). Concurrently, the study discovered that the rapid influx of Ca²^+^ from the extracellular matrix into mitochondria, releasing ROS, promotes RIPK1/RIPK3-dependent necroptosis (55). Emerging evidence indicates a close association between mitochondrial function-related proteins—mitochondrial phosphatase Phosphoglycerate mutase 5 (PGAM5) and Drp1—and necroptosis. Drp1 downregulation-mediated mitochondrial fragmentation inhibits TNF-induced necroptosis in HeLa and HT-29 cells (56). PGAM5 has also been found to act at key points in multiple necroptosis-related death pathways (56). Research by Zhou et al. demonstrated that downstream of RIPK1/RIPK3/MLKL, PGAM5 dephosphorylates Drp1, inducing mitochondrial fragmentation and promoting necroptosis (57). Furthermore, PGAM5, acting downstream of RIP3/MLKL, can also activate CypD to regulate necroptosis (58). Downregulation of mitochondrial fusion proteins Mfn1/2 and OPA1 can prevent classical necroptosis by reducing TAK1-mediated RIP1-Ser166 phosphorylation (59). A recent study revealed that mPTP opening is also associated with cellular necroptosis; RIPK3 can activate CaMKII, which phosphorylates the mitochondrial calcium uniporter (MCU), leading to mitochondrial Ca²^+^ overload. This triggers mPTP opening, generating excessive ROS and promoting necroptosis (60, 61). Research by Liu et al. found that regulating mtDNA can prevent necroptosis in post-mitotic cardiomyocytes (62). The mechanism by which mtDNA promotes necroptosis may involve activating the mtDNA-STING signaling pathway. However, another study suggested that mitochondria are not essential for necroptosis (63). Tait SW et al. specifically removed mitochondria via mitophagy and induced RIPK3 activation; the results showed that extensive mitochondrial depletion through mitophagy did not affect necroptosis (64). Therefore, mitochondria are important players in necroptosis under certain conditions, but they are not absolutely essential.

### 3.3 Mitochondria-driven necroptosis as a key pathway in the development of OP

Cells in bone tissue contain bone marrow mesenchymal stem cells, osteoblasts, osteoclasts, osteocytes, etc., which are involved in the formation and destruction of bone and are critical for the maintenance of bone homeostasis. Several studies have confirmed that Necroptosis may be an important target for restoring bone mass in OP characterized by bone loss, altered bone microstructure, and increased bone fragility. Studies have found that necrotitic apoptosis is one of the key factors in bone cell death and bone loss in ovariectomy (OVX) induced OP (65), and specific inhibitors of necrotitic apoptosis can prevent bone loss in estrogen deficiency (66). Chronic heavy alcohol drinking can also regulate the Necroptosis of osteoblasts to induce osteopenia. Numerous studies have confirmed that the involvement of mitochondria in regulating Necroptosis of various bone-related cells, which then affects OP disease progression, such as in bone cells, Zhang et al. found that mtROS can also mediate necroptotic apoptosis of osteocytes through the activation of the JNK/IL-18 pathway (67). In the osteoblasts, Tian et al. found that Necroptosis in osteoblasts is similarly regulated by mitochondria, Reactive oxygen species (Reactive Oxygen Species, Packaging of ROS) can trigger the mPTP open one’s mouth, Causing a necrotizing apoptotic pattern of osteoblasts (68), While Feng et al. found that during dexamethasone-induced anterior osteoblast cell death, Necroptosis and apoptosis are accompanied by reciprocal changes in mitochondrial function (69). In BMSCs, RIPK1 deficiency causes mPTP opening, mtDNA damage, mitochondrial dysfunction, and thus induced Necroptosis in BMSCs (70), while another study confirmed that BMSCs-derived mitochondria can effectively ameliorate Necroptosis in other damaged cells (71). In addition, mitochondria can also affect the Necroptosis of other cells in the bone marrow. For example, Wei et al. found that mouse monocyte macrophages can differentiate into osteoclasts under the action of cigarette smoke extract, the change of mitochondrial function leads to the occurrence of Necroptosis of RAW 264.7 cells (72). Spencer et al. showed that RAW 264.7 cells treated with LPS (Lipopolysaccharide) and Z-VAD could release mitochondria-containing Microparticles (MPs) to induce further cell death (73). Multiple studies demonstrate that intervertebral disc degeneration and osteoporosis are bidirectionally correlated and mutually reinforcing (74, 75), with nucleus pulposus (NP) cells serving as key cellular mediators in this process. Research indicates that in bone disease-associated NP cells, ROS, the respiratory chain substrates of the inner mitochondrial membrane, regulates ER stress and “endoplasmic reticulum-mitochondrial Ca^2+^ crosstalk” promotes compression-induced Necroptosis of rat nucleus pulposus cells (76). Although many of the above studies have proved that mitochondria regulate Necroptosis in bone-related cells (**Supplementary Table 2-1**), there are relatively few further studies on them in OP and still need further exploration.

### 3.4 Inhibiting mitochondrial necroptosis: a therapeutic strategy for bone-related diseases

Currently, it has been demonstrated that targeting mitochondria can regulate the Necroptosis pathway of BMSCs and bone cells involved in the OP disease process (**Supplementary Table 2-2**). The study by Tian et al. revealed that RIPK1 enhances proliferation, differentiation, and migration of BMSCs in OP by maintaining mitochondrial homeostasis via p53(Tumor protein 53), thereby suppressing necroptosis (70), Feng found that drugs associated with necroptosis inhibitor Necrostatin-1, Can ameliorate the necrotic morphology and mitochondrial lesions exhibited by bone cells, accelerated glucocorticoid-induced bone formation in OP rats (77). However, there are relatively few studies targeting mitochondria to regulate Necroptosis to improve OP, and more studies reveal the mechanism by which targeting mitochondria regulates necrotizing apoptosis to improve the process of other Musculoskeletal-related diseases. For example, in intervertebral disc degeneration, the inhibition of MyD 88 signal can effectively reduce the ROS level and ATP level, change the mitochondrial ultrastructure, and rescue the Necroptosis of nucleus pulposus cells (78). Mitochondria are an important source of ROS, and studies have found that targeting oxidative stress with amobarbital(Amo), can reduce cell Necroptosis and prevent intervertebral disc degeneration (79). RIPK1-mediated mitochondrial dysfunction and oxidative stress induced Necroptosis of nucleus pulposus cells during compressive injury, while the apoptosis inhibitor necrostatin-1 (Necrostatin-1, Nec-1) and mPTP inhibitor cyclosporinA (CsA) can restore the mitochondrial function of NP cells and reduce their oxidative stress status and improve Necroptosis (80). Further studies revealed that the synergistic utilization of Necrostatin-1 and Z-VAD-FMK improved compression-mediated Necroptosis of nucleus pulposus cells by restoring mitochondrial function (81). In osteoarthritis (OA), mitochondrial transplantation can enhance ATP synthesis, oxygen consumption and cell viability, and improve the Necroptosis of chondrocytes (82). In spinal cord injury (SCI), necrostatin-1 can reduce mitochondrial dysfunction and promote cytoprotection and physiological function after SCI, mainly mitochondria-mediated (83, 84). Inflammation can increase bone resorption and reduce bone formation, and patients with chronic inflammatory diseases have a higher risk of OP. In inflammatory conditions, LPS protects against hydrogen peroxide-induced BMDMs from PARP-1-dependent death by downregulating PARP-1 (Polymerase-1) expression, upregulation of antioxidant proteins and the metabolic transition from mitochondrial respiration to aerobic glycolysis (85).

## 4 Pyroptosis and OP

### 4.1 Definition of pyroptosis

Charptosis, also known as cell inflammatory necrosis. Unlike apoptosis, pyroptosis is characterized by cell expansion causing membrane rupture, which in turn leads to the release of cytokines and the activation of a series of cascades (86). Pyroptosis is a programmed cell death mediated by the assembly of the inflammasome, accompanied by the cleavage of the gasdermin D (GSDMD) family proteins and the release of proinflammatory cytokines, in which GSDMD is a key process to stimulate pyroptosis (87, 88). In the classical pyroptosis pathway, pathogen-related molecular pattern (PAMP) or cell-derived damage-associated molecular pattern, (DAMPs) into the cytosolic cytosol, Initiate the assembly of the activated NLRP 3 (NOD-like receptor thermal protein domain associated protein 3) inflammasome. Subsequently, the inflammasome proteins oligomerize and act through the recruitment of apoptosis-associated speck-like protein (ASC) to continue forming ASC oligomers, activating inflammatory cysteine protease caspase-1 to cleave GSDMD and the cytokine pro-IL-1 β, pro-IL-18.Next, the cleaved GSDMD-N domain binds to phosphoinositides (PIs) in the plasma membrane and oligomerizes to form a nonselective pore penetrating the cell membrane, releasing the cell content and the entire inflammasome complex to the outside, finally leading to water flow into the cell, cell lysis and pyroptosis (89). In the non-classical pyroptosis pathway, cell pyroptosis is associated with various biomolecules, such as caspase and granzyme. Cytosolic inflammatory factors such as LPS can activate mouse caspase-11 and cleave GSDMD, thereby triggering pyroptosis. GSDMD can also be cleaved by active caspase-8, which ultimately mediate the assembly of NLRP 3 inflammasome and the cleavage of IL-1 β and IL-18 precursors, triggering pyroptosis.Activated caspase-11 and caspase-3 can also induce cell pyroptosis. In the granzyme-mediated pathway, killer T cells (natural killer T cells) can trigger extensive target cell pyroptosis by directly cleaving and activating granzyme A (GzmA) and granzyme B granzyme B (GzmB) and GSDME, respectively. GzmB can also indirectly activate GSDME to trigger cell death via the cleavage of caspase-3 (90). Pyroptosis is believed to be widely involved in the development of various metabolic diseases such as OP.

### 4.2 Mitochondrial molecular mechanisms in pyroptosis: synergistic effects and homeostatic imbalance

**Figure 3:**
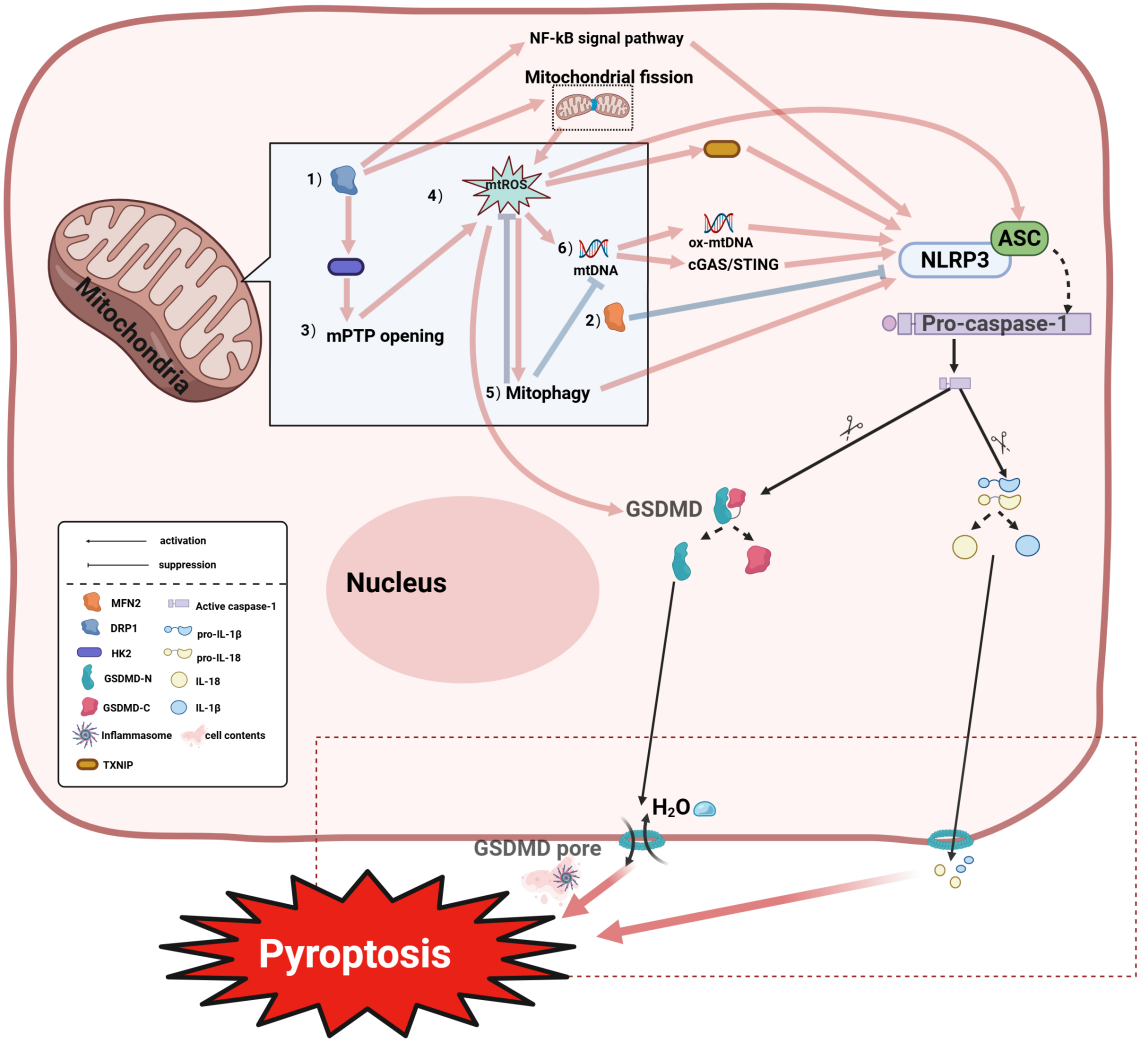
Mitochondria coordinately regulate pyroptosis through multiple mechanisms:(1) DRP1 promotes NLRP3 (NOD-like Receptor Pyrin Domain Containing 3) inflammasome activation via enhancing mtROS accumulation, mPTP opening, and NF-κB pathway stimulation; (2) MFN2-mediated mitochondrial fusion antagonizes the aforementioned processes; (3) The interaction between DRP1 and Hexokinase 2 (HK2) regulates mPTP opening; disruption of this interaction promotes pathological mPTP opening, exacerbating mtROS accumulation and pyroptosis; (4) The mtROS cascade: Mitochondrial ROS (mtROS) generated by respiratory chain inhibition activates NLRP3-dependent pyroptosis through: inducing autophagy; enhancing TXNIP-NLRP3 interaction; and inducing oxidized mtDNA (ox-mtDNA) release which further activates NLRP3. Additionally, mtROS can promote pyroptosis by inducing oxidation of GSDMD; (5) Mitophagy regulation: Mitophagy exhibits dual modulation of pyroptosis—mtROS upregulation may enhance mitophagy to promote NLRP3 activation, whereas damaged mitochondria clearance via mitophagy suppresses inflammasome assembly by inhibiting mtROS/mtDNA release; (6) mtDNA signaling: Released ox-mtDNA directly binds and activates NLRP3 to trigger pyroptosis; alternatively, mtDNA activates the cGAS-STING pathway, amplifying inflammatory responses that promote pyroptosis . IL-18, Interleukin-18; IL-1β, Interleukin-1 beta; pro-IL-18, Pro-Interleukin-18; pro-IL-1β, Pro-Interleukin-1 beta; HK2, Hexokinase 2; GSDMD-N, Gasdermin D N-terminal domain; GSDMD-C, Gasdermin D C-terminal domain.

As the initiation factor of pyroptosis, mitochondria are able to induce pyroptosis through various pathways (**Figure 3**). Mitochondria can influence NLRP3 activation through dynamic changes. Mitochondrial dynamics refers to the process by which mitochondria maintain the dynamic balance of the mitochondrial network through continuous fission and fusion. Mitochondrial fission is mediated by fission proteins, enabling the separation of healthy and damaged mitochondria and mitochondrial distribution during mitosis (91), regulated by the key factor DRP1 which can be recruited to form active fission sites on the outer mitochondrial membrane (OMM) to promote fission. Mitochondrial fusion plays a crucial role in energy exchange between mitochondria and maintaining mitochondrial morphology/functional integrity, primarily mediated by Mitofusin 1 (MFN1), MFN2 and OPA1 (91). MFN2 serves as the core regulatory protein for OMM fusion, localizing to the OMM as a key factor in fusion and morphology maintenance (92); MFN2 mutations cause fusion defects while MFN1/2-deficient cells exhibit severe fragmentation (93). Zou et al. demonstrated that DRP1 activates the NLRP3 inflammasome by promoting fission, exacerbating pyroptosis, whereas inhibiting DRP1 phosphorylation and upregulating fusion-related MFN2 blocks NLRP3 activation, reducing GSDMD-mediated pyroptosis and IL-1β release (94). Further research suggests that DRP1’s activation of NLRP3 may be linked to its mechanism of inducing excessive mitochondrial fission, which forms damaged mitochondria, thereby promoting the accumulation of mtROS that activates NLRP3 (95). Additionally, DRP1 directly interacts with mPTP-associated hexokinase 2 (HK2), causing excessive mPTP opening that exacerbates mtROS accumulation and pyroptosis (96). DRP1 also activates NLRP3 via inflammation-related pathways like NF-κB (97). mtROS and mitophagy regulate NLRP3 activity (98). mtROS can inhibit respiratory chain complexes I/III generate mtROS promoting spontaneous NLRP3 activation (99). mtROS acts as a key NLRP3 activator through multiple mechanisms—oxidatively modifying GSDMD (90), releasing oxidized mtDNA (ox-mtDNA) after mitochondrial damage to activate NLRP3 (100, 101), promoting ASC oligomerization into pyroptosomes, and disrupting TXNIP-Thioredoxin(TRX) interactions while enhancing TXNIP-NLRP3 binding (102). NLRP3 activation conversely promotes mtROS generation (103). Mitophagy regulation also inhibits NLRP3-induced pyroptosis (104), though its role is context-dependent: impaired mitophagy causes persistent mtROS-producing mitochondria driving spontaneous inflammasome activation (99), while damaged mitophagy activates mtDNA/cGAS/STING signaling promoting hepatocyte pyroptosis (103). Notably, mtROS scavengers alleviated upregulated ox-mtDNA and mitophagy to suppress NLRP3 activation in NaAsO_2_-induced hepatic insulin resistance models (105). Additional studies indicate that excessive Ca²^+^ mobilization triggers mitochondrial damage, releasing mtDNA and thereby promoting NLRP3 inflammasome activation (106). Oxidized mtDNA (ox-mtDNA) can directly bind to and activate NLRP3 (107). Alternatively, mtDNA released into the cytosol may activate NLRP3 by triggering the cGAS-STING signaling pathway (108). As mentioned above, mitochondria are associated with cell ferroptosis, and mitochondria can affect cell ferroptosis through a variety of pathways.

### 4.3 Mitochondria-regulated pyroptosis in the pathogenesis of OP

Several studies have proved that pyroptosis and its inducing factors influence the occurrence and development of OP. Proptosis inducing factor NLRP 3 is a key participant in osteoporosis, Yang et al. confirmed that knockdown of NLRP 3 can inhibit pyroptosis, improve the inflammatory status of bone tissue and promote osteogenic differentiation of osteoblasts (109), while the overexpression of deubiquitination enzyme USP 1 can also play a protective effect on MC3T1-E3 (110). In addition, Li et al. found that by activating Ca^2+^ influx, it affects mitochondrial dynamics and can inhibit NLRP 3 inflammasome activation, thus regulating the osteogenic effect of BMMCs (111). GSDMD, a key factor of pyroptosis, can play an important role in regulating bone homeostasis by regulating osteoclast function (112). Several studies have found that mitochondria play an important regulatory role in a types of OP-associated pyroptosis. Liu et al. found that lncRNA H19 could inhibit LDL-induced NF- κ B activation, leading to mitochondrial dysfunction and reducing the production of reactive oxygen species (ROS), thus playing a role in cell pyroptosis (113). Lysosomal disruptors (Apilimod) can activate the NLRP 3 inflammasome in the BMDMs through lysosomal (Lysosomes) -mediated mitochondrial damage (114). Another study found that NLRP 3 inflammasome activation and pyroptosis inhibition in BMDMs were achieved by promoting mitophagy (115). GSDMD targeting the plasma membrane in BMDMs promotes rapid mitochondrial collapse as well as the initial accumulation of mitochondrial DNA in the cytoplasm, and then promotes DNA release from cells in the event of plasma membrane rupture to complete the process of pyroptosis (116). In bone-like cells MLO-Y4, bisphenol A (BPA) can induce pyroptosis of MLO-Y4 cells through the ROS/NLRP 3/Caspase-1 pathway (117). In the development of bone, osteolipid differentiation imbalance is an important cause of OP, and the function of adipocytes may be associated with mitochondria-mediated cell pyroptosis, Qian, et al., found that bone morphogenetic protein type II receptor (BMPR 2) knockout can regulate mitochondria and induce adipocyte pyroptosis and inflammation level (118). Alternatively, in NP cells, mitochondrial DNA can induce NP cell pyroptosis through the TLR 9-NF- κ B-NLRP 3 axis (119). MFN2 and DRP1 play crucial roles in maintaining bone homeostasis (120–122) and preserving the function of relevant bone cells MFN2 levels change during osteoblast differentiation, and MFN2 conditional knockout in 8-week-old female mice showed a mild increase in cortical bone; by 30 weeks of age, both bone volume fraction and cortical bone were significantly increased (123). Research by Yao et al. found that regulating mitochondrial dynamics in mesenchymal stem cells and upregulating levels of dynamics-related proteins like MFN2 can promote cell proliferation and osteogenic differentiation capacity (121). Additionally, MFN2 plays a key role in regulating pyroptosis and NLRP3 inflammasome activation in NP cells: MFN2-knockout NP cells showed reduced Lactate dehydrogenase (LDH) release, while MFN2-overexpressing NP cells exhibited increased Caspase-1 activation, enhanced IL-1β cleavage, and elevated NLRP3 protein and mRNA expression levels, indicating that MFN2 can promote NLPR3 expression and activate the NLRP3 inflammasome, leading to NP cell pyroptosis (124). These studies demonstrate that mitochondria regulate pyroptosis or the expression of pyroptosis-inducing factors in relevant bone cells, which may influence the development of osteoporosis or other bone-related diseases (**Supplementary Table 3-1**).

### 4.4 Targeting mitochondria to suppress pyroptosis: a therapeutic strategy for bone disorders

At present, existing studies have confirmed that targeting mitochondria can become a therapeutic strategy to effectively regulate pyroptosis involved in osteogenesis process, such as Wang et al., found that BMSCs can be used as mitochondrial donor cells, the mitochondria donated to blast lasts, thus reducing the mtROS produced by mitochondria, and reduce the resulting NLRP 3 inflammasome induced odontoblasts pyroptosis (125). However, more studies have focused on other bone-related diseases, such as in myelodysplastic syndromes (MDSs), where abnormal hematopoietic stem cells and progenitor cells (HSPCs) affect the activation of inflammasomes by releasing oxidized mitochondrial DNA (ox-mtDNA) (126). In spinal cord injury, the novel mitochondria-targeted peptide elamipretide (EPT) can inhibit NLRP 3 inflammasome activation and pyroptosis in spinal cord tissue, and promote the functional recovery after spinal cord injury (127). Liang’s study showed that the mitochondrial antioxidant SS-31 could inhibit LPS-induced pyroptosis and inflammation in NP cells by scavenging ROS and maintaining the stability of mitochondrial dynamics (128). In multiple myeloma (Multiple Myeloma, MM)), proteasome inhibitor PIs can trigger pyroptosis of myeloma cells (Myeloma cells) through the mitochondrial BAX/GSDME pathway and improve the therapeutic efficiency of multiple myeloma treatment (129). In the treatment of mitochondria-targeting polymer micelle(OPDEA-PDCA), targeting mitochondria, can induce osteosarcoma cell pyroptosis by initiating mitochondrial oxidative stress (115). In some other inflammation-related models, drugs can inhibit the activation of pyroptosis or pyroptosis-related inducers by regulating or protecting mitochondrial homeostasis in BMDMs. For example, bergamot can inhibit NLRP 3 inflammasome activation and pyroptosis in BMDMs by promoting mitophagy and maintaining mitochondrial homeostasis (130). While another study found that TRPV 4 knockdown could ameliorate mitochondrial dysfunction and alleviate mtROS-mediated cell pyroptosis and cartilage degeneration through the DRP1-HK2 axis (96). Reduction of PIM-1 can inhibit mitochondrial reactive oxygen species/chloride intracellular channel proteins (mtROS/CLICs) in synovial macrophages, eventually leading to ASC oligomerization, blocking the activation of the NLRP 3 inflammasome, and relieving osteoarthritis (131). In rheumatoid arthritis, leakage of mtDNA in MRE11A low-expressing T cells triggers the inflammasome, causing activation of caspase-1, release of IL-1 β, triggering pyroptosis, while MRE11A-overexpressing T cells can reduce synovial inflammation in this model (132). In gouty arthritis, resveratrol can promote mitochondrial autophagy in BMDM through the activation of the Pink1/Parkin pathway, and subsequently inhibit the activation of the NLRP 3 inflammasome (133). Feng et al. found that the natural compound Scoparone, isolated from the Chinese herbal Artemisia capillary, could block NLRP 3 inflammasome activation by enhancing mitophagy in BMDM, thus showing effective anti-inflammatory effects and treating inflammasome-related diseases (134). 1 ′ -acetyloxycholitol acetate prevents pyroptosis by inhibiting inflammasome activation through mitochondrial ROS, mtROS, in mouse BMDM (135). Stomatin-like protein-2 (SLP-2) can attenuate BMDM cell pyroptosis by protecting mitochondrial function under hypoxia (136). The above research indicates that targeting mitochondria for pyroptosis intervention is an effective therapeutic strategy in skeletal system disorders (as summarized in **Supplementary Table 3-2**); however, studies on its role in osteoporosis remain limited and require further experimental validation.

## 5 Ferroptosis and OP

### 5.1 Definition of ferroptosis

Ferroptosis is a novel, iron-dependent, non-apoptotic form of programmed cell death (137). It is characterized by the accumulation of iron-dependent lipid peroxides (138), which leads to the change of cell mitochondrial morphology, the accumulation of reactive oxygen radicals, cellular membrane rupture, and iron ions participate in this process. Under normal conditions, lipoxygenase usually oxidizes polyunsaturated fatty acids (PUFA), but the lipid repair Recombinant Glutathione Peroxidase 4(GPX 4) and its cofactor glutathione (GSH) lead to a rapid decrease in the level of PUFAs oxidized by lipoxygenase. The ferroptosis process is caused by the inhibition of the cystine-glutamate transport receptor System Xc, which can transport glutamicacid out of cells and transport cystine into cells to synthesize GSH. The inhibition of System Xc will further lead to the reduction of GSH biosynthesis and the inactivation of GPX 4. Subsequently, the cells die due to the overwhelming lipid peroxidation (LPO) (139–142). Inhibition of the GPX 4 enzyme can also directly lead to this effect. But ferroptosis is essentially a flexible mechanism, and dozens of metabolites and proteins can be involved in the regulation of toxic accumulation of membrane lipid peroxides, including those involved metabolites and protein (143). However, the two main initiation pathways include: exogenous or transporter-dependent pathway (e. g. System Xc) and endogenous or enzyme regulation pathway (e. g. GPX 4) (144). In addition, ferroptosis suppressor protein-1(FSP 1) has been proposed to be another important way to affect ferroptosis. FSP 1 can prevent lipid oxidation to inhibit cell ferroptosis by reducing CoQ 10.

### 5.2 Mitochondrial damage-induced ferroptosis

**Figure 4:**
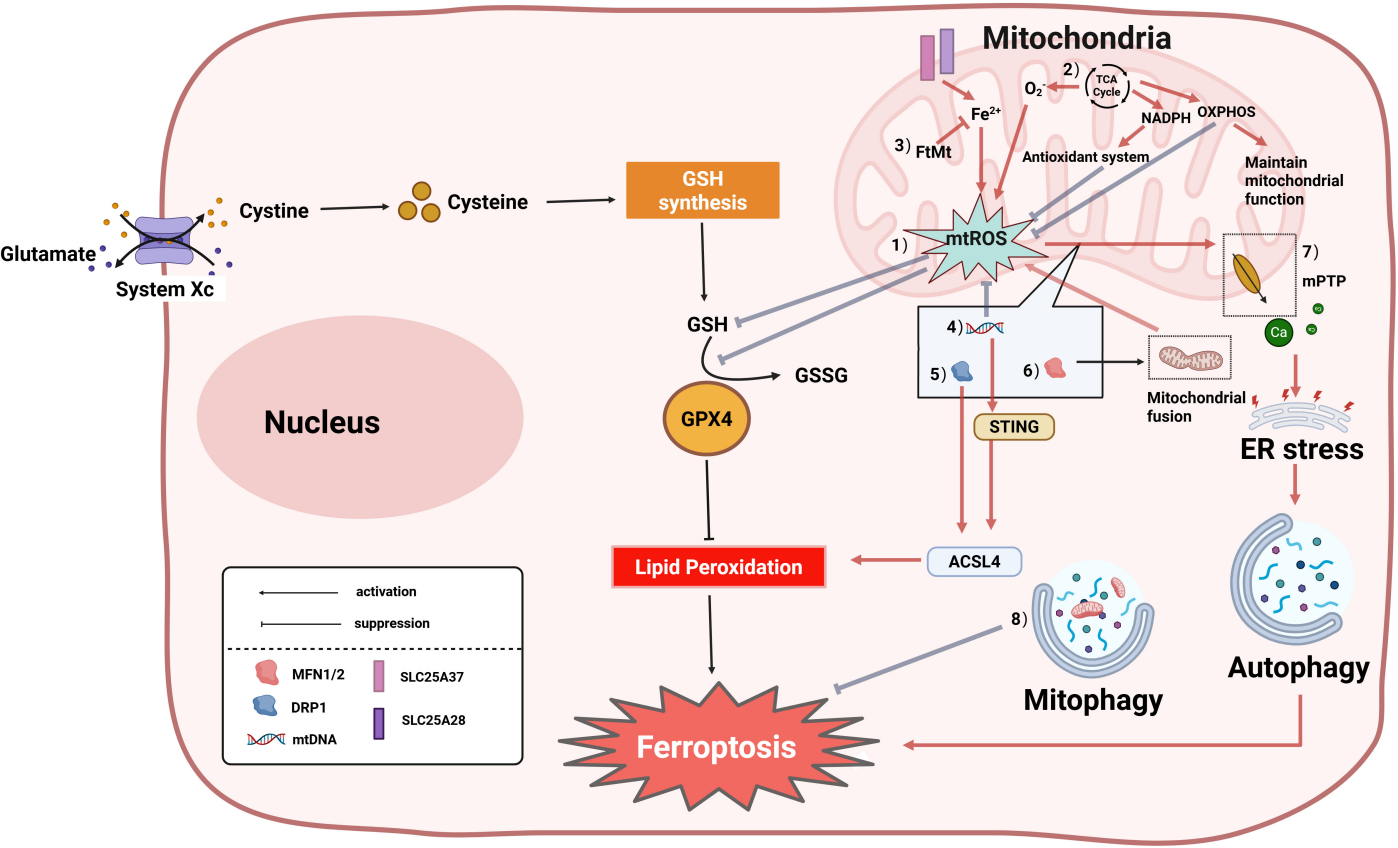
Core molecular mechanisms of mitochondrial regulation of ferroptosis. Ferroptosis is accompanied by alterations in mitochondrial morphology, and mitochondria can also regulate cellular ferroptosis through multiple pathways. The specific mechanisms are as follows:(1) Research indicates that the mechanism of mitochondrial damage-induced ferroptosis differs from traditional pathways; the primary mechanism may be cellular ROS accumulation exceeding the redox capacity maintained by GSH and GSH-based phospholipid hydroperoxidases; (2) The tricarboxylic acid (TCA) cycle drives ferroptosis by promoting mtROS generation; electron transport chain (ETC) dysfunction can also reduce NADPH synthesis, weaken the antioxidant defense system, and promote cellular ferroptosis; (3) Mitochondrial Ferritin (FtMt): Chelates free iron ions (Fe²^+^), inhibiting iron-dependent lipid peroxidation; (4) mtDNA abnormalities: Trigger ferroptosis via the STING-Acyl-CoA Synthetase Long Chain Family Member 4 (ACSL4) pathway or by promoting mtROS accumulation, leading to GSH depletion; (5) DRP1 dephosphorylation promotes mitochondrial fission, activates ACSL4, and accelerates ferroptosis; (6) Mitofusin 1/2 (MFN1/2)-mediated mitochondrial fusion increases mtROS generation, inducing ferroptosis; (7) mPTP: mPTP opening causes Ca²^+^ efflux and endoplasmic reticulum (ER) stress, ultimately promoting autophagy-dependent ferroptosis; (8) Mitophagy suppresses ferroptosis by clearing damaged mitochondria. GSSG, Glutathione Disulfide; GPX4, Glutathione Peroxidase 4; SLC25A37, Solute Carrier Family 25 Member 37/Mitoferrin-1; SLC25A28, Solute Carrier Family 25 Member 28/Mitoferrin-2.

Ferroptosis, as a novel form of PCD, is often accompanied by mitochondrial morphological changes, including reduced mitochondrial volume, rupture of the OMM, and decreased or absent mitochondrial cristae (141). Previous studies suggested ferroptosis did not require mitochondrial involvement (139); however, recent research increasingly reveals that mitochondria play a key role primarily in cysteine deprivation-induced ferroptosis, but have a limited role in glutathione Peroxidase 4 (GPX4) inhibition-induced ferroptosis. In-depth studies demonstrate that mitochondria participate in ferroptosis regulation through multiple molecular mechanisms, primarily involving mtROS, the tricarboxylic acid (TCA) cycle, oxidative phosphorylation (OXPHOS), iron ion regulation, mtDNA, and mitochondrial dynamics (145) (**Figure 4**). Research indicates that the mechanism of mitochondrial damage-induced ferroptosis differs from traditional pathways; the main mechanism may be cellular ROS accumulation exceeding the redox capacity maintained by GSH and GSH-based phospholipid hydroperoxidases, where the burst of lethal mtROS and accumulation of lipid peroxidation products affect iron metabolism-related proteins in mitochondrial membranes, serving as the primary cause mediating neuronal ferroptosis (146, 147). The mitochondrial TCA cycle and electron transport chain(ETC) promote ferroptosis by mediating lipid peroxide production. ETC dysfunction can also reduce NADPH synthesis, weaken the antioxidant defense system, and promote cellular ferroptosis (145). Mitochondrial OXPHOS is a core metabolic pathway for ATP generation via the ETC and ATP synthase; OXPHOS damage causes mitochondrial membrane potential collapse and excessive mtROS accumulation, while restoring OXPHOS complexes can recover membrane potential, improve mitochondrial function, and antagonize ferroptosis (148). Mitochondria regulate iron homeostasis via mitochondrial ferritin (FtMt). After iron ions enter the cell extracellularly, they can access mitochondria via solute carrier family 25 member 37/mitoferrin-1(SLC25A37) and solute carrier family 25 member 28/mitoferrin-2(SLC25A28), and mitochondria regulate cellular iron metabolism through FtMt. FtMt prevents iron-induced oxidative stress ferroptosis by chelating excess free iron ions (149). Multiple studies indicate mtDNA involvement in ferroptosis: mtDNA depletion in hepatocytes of patients causes mitochondrial dysfunction, reduced ATP production, and enhanced ROS, leading to GSH depletion-induced ferroptosis (150). mtDNA leakage in HK-2 cells increases STING expression and interacts with acyl-CoA synthetase long chain family member 4(ACSL4), inducing ACSL4-dependent ferroptosis (151). At the dynamics regulation level, DRP1-mediated mitochondrial fission and Mfn1/2-regulated fusion processes affect ferroptosis sensitivity through different mechanisms. Miao et al. reported that Hsp90 overexpression and calcineurin (CN)-mediated dephosphorylation of DRP1 at serine 637 (Ser637) promote ferroptosis by altering mitochondrial morphology and increasing ACSL4-mediated lipid peroxidation (152). Mitochondrial MFN1/2 can also form complexes with STING, mediating mtROS production via mitochondrial fusion, leading to lipid peroxidation and promoting ferroptosis (153). Additionally, ROS release activates the mPTP, causing Ca²^+^ release and endoplasmic reticulum (ER) stress-induced autophagy-dependent ferroptosis (154). Another study showed that activating mitophagy enhances cellular resistance to ferroptosis by clearing damaged mitochondria (146). However, it is particularly noteworthy that the role of mitochondria in ferroptosis may be context-dependent. Studies indicate that inhibiting mitochondrial function effectively blocks cysteine deprivation-induced ferroptosis, but in GPX4 inactivation models, cells can still undergo ferroptosis independently of mitochondria (146). This difference suggests that ferroptosis may involve heterogeneous molecular mechanisms under different induction conditions, and its specific regulatory network requires further elucidation.

### 5.3 Mitochondria-mediated ferroptosis in the development of OP

Increasing evidence suggests that ferroptosis plays an important role in the course of OP. Iron overload reduces the levels of superoxide dismutase and glutathione, increases the generation of reactive oxygen species, triggers lipid peroxidation, increases the expression of ferroptosis-related proteins, and induces ultrastructural changes in mitochondria, reduces osteoblasts viability and suppresses their *in vitro* osteogenic differentiation and mineralization. Therefore, maintaining iron homeostasis and targeting ferroptosis in osteoblasts are considered a potential measure for the treatment or prevention of iron overload-induced OP (155). Further studies show that mitochondrial reactive oxygen species, mitochondrial dysfunction, mitochondrial autophagy, Mitochondrial dynamics and other mechanisms mediating the occurrence of cell ferroptosis are closely related to the disease progression of OP (**Supplementary Table 4-1**). Jin et al. found improved mitochondrial dysfunction and suppressed BMSCs ferroptosis in OP in type 2 diabetes by regulation of the AMPK/SIRT 1 pathway (156). Ferritin, mitochondrial(Ft Mt) is a protein that stores iron ions and blocks toxic iron ions in mitochondria. Wang et al. showed that Ft knockdown can promote mitophagy ferroptosis in type 2 diabetic osteoblasts, the study also confirmed the classic mitophagy and oxidative stress related pathways to induce mitophagy can participate in the regulation of ferroptosis in osteoblasts (157). Research by Yao et al. indicates that regulating mitochondrial fusion and fission, as well as the PI3K/AKT/mTOR and MAPK pathways, can effectively alleviate iron overload-induced inhibition of proliferation and osteogenic differentiation in BMSCs (121). Thus, targeting mitochondria to ameliorate cellular ferroptosis may serve as an effective strategy for treating osteoporosis.

### 5.4 Targeting mitochondria to inhibit ferroptosis: a therapeutic strategy for bone- related diseases

Mitochondria-related ferroptosis plays an important role in OP and related diseases, and targeting mitochondria to regulate ferroptosis has become one of the important strategies to study the therapeutic drugs for OP and related diseases (**Supplementary Table 4-2**). First, the occurrence of ferroptosis is necessarily accompanied by ultrastructural changes in the mitochondria. In addition, it was found that the iron chelators deferriamine and siristatin-1 can significantly alleviate the negative effects of ferroptosis in osteoblasts, improve the ultrastructure of mitochondria, and promote bone formation in the *in vivo* model of OP (155). Melatonin can also improve the morphological changes of mitochondria by activating the Nrf 2/HO-1 pathway associated with oxidative stress *in vitro* and *in vivo*, and significantly reducing the level of ferroptosis in osteoblasts (158). Meanwhile, it have found that changing mitophagy, regulating reactive oxygen species in mitochondria and improving mitochondrial function can inhibit ferroptosis in OP. Mitochondrial ferritin FtMt can inhibit the occurrence of ferroptosis in osteoblasts by reducing the oxidative stress caused by excess ferrous iron ions through mitophagy (157). Mitochondria are the main source of reactive oxygen species in cells, and studies have shown that antioxidants are able to prevent ferroptosis by reducing ROS and LPO production (159). In a rat model of OP with bilateral oophorectomy, the intestinal flora affected the *de novo* synthesis of glutathione (GSH), a key factor for ferroptosis, by inhibiting mitochondrial biological activity and ROS accumulation by regulating its key glutamate-cysteine ligase catalytic subunit (Gclc) and the cAMP response element-binding(CREB) pathway (160). In many other correlated diseases of the skeletal system, mitochondria have also been used as targets for the study of novel drugs regulating cell ferroptosis in diseases. B-BMSCs pretreated with δ -tocotrienol promotes wound healing ability by inhibiting BACH 1-related (The BTB and CNC homology 1) ferroptosis (161). In femoral head necrosis, exogenous melatonin can inhibit BMSCs ferroptosis, maintain cell activity, and reduce bone loss in rats with femoral head necrosis by improving mitochondrial damage and cellular lipid peroxidation (162). Oxidative stress is an important cause of cell ferroptosis. Studies have shown that actin can reduce cell oxidative stress damage by enhancing osteoblast antioxidant defense and mitochondrial biogenesis (160). In a cellular model of age-related bone loss, sciadopitysin can protect mitochondria from oxidative stress damage by preventing oxidochondrial membrane potential dissipation, loss of ATP, and release of ROS (163).

## 6 Autophagy and OP

### 6.1 Definition of autophagy

Autophagy is a conserved lysosomal degradation pathway in which cellular components are dynamically degraded and re-processed to maintain physical homeostasis (164). Autophagy can be roughly divided into three types: macroautophagy, chaperone-mediated autophagy (CMA), and microautophagy. Macroautophagy is recognized as the main autophagy type and a key homeostatic pathway that promotes the degradation and recycling of cellular material (165). In the process of macroautophagy, autophagy-related proteins (ATG) form double membrane vesicles, called autophagosome, by coordinating with ULK 1 complex and class III PI3K kinase complex, and their wrapped cell contents fuse with the lysosome, leading to the degradation of through the activity of lysosomal hydrolase (166). cargo recruitment of macroautophagy can be divided into non-selective and selective depending on whether the degraded substrate is specific. Macroautophagy can be divided into non-selective autophagy and bulk autophagy and selective autophagy. Non-selective autophagy occurs in a non-selective manner, including random intake of cytoplasmic material for degradation. Non-selective autophagy mainly plays an important role in cell starvation, providing amino acids for cells to meet the nutritional requirements (165). Selective autophagy, regulated by autophagic cargo receptors, binds to specific cargo that have been degraded by ubiquitin-dependent or related processes, selectively degrades specific cell components, mainly used to protect cell structure (167). Chaperone-mediated autophagy and microautophagy do not require the activity of ATG proteins, in which delivery of cargo to the lysosome depends on the activity of the chaperone and invagination of the lysosomal membrane to encapsulate the cellular material, respectively (115).

### 6.2 Mitophagy as a critical mechanism of selective autophagy

**Figure 5:**
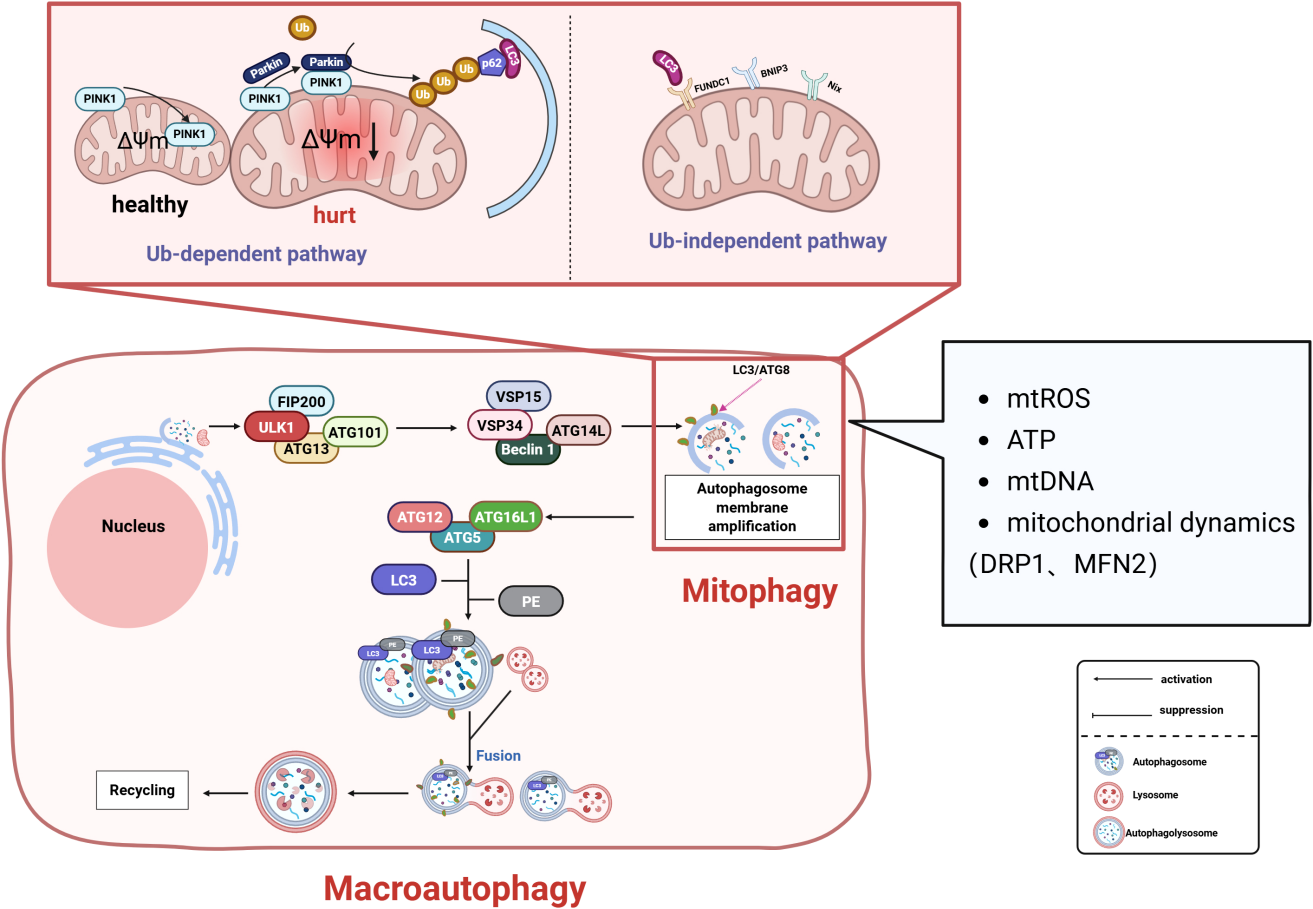
Mitophagy regulates mitochondrial quality control through dual pathways to maintain cellular homeostasis: Ubiquitin-Dependent Pathway: During mitochondrial depolarization, PTEN-induced Putative Kinase 1 (PINK1) accumulates on the outer mitochondrial membrane (OMM), recruiting and activating Parkin. This mediates ubiquitination of OMM proteins. Autophagy adaptor proteins (e.g., p62/SQSTM1) recognize ubiquitinated substrates and bind Microtubule-associated protein 1A/1B-light chain 3 (LC3), facilitating autophagosome formation. Receptor-Dependent Pathway: Under hypoxia or stress conditions, receptors including FUN14 Domain Containing 1 (FUNDC1) and BCL2/Adenovirus E1B 19 kDa Interacting Protein 3-Like (NIX/BNIP3L) undergo dephosphorylation. These receptors directly interact with LC3 to initiate autophagosomal engulfment of damaged mitochondria. Regulatory Network: mtROS, ATP levels, mtDNA, and mitochondrial dynamics (fission/fusion balance) collectively regulate mitophagy. These factors synergistically determine mitochondrial health and cellular viability. FIP200, Focal Adhesion Kinase Family Interacting Protein of 200 kDa; ATG, Autophagy-related protein; ULK1, Unc-51 Like Autophagy Activating Kinase 1; VPS, Vacuolar Protein Sorting; PE, Phosphatidylethanolamine; p62/SQSTM1, Sequestosome 1.

Mitochondria are organelles within cells and serve as the central hub for energy metabolism in eukaryotic cells. When mitochondria are damaged, they release harmful substances that threaten cell survival. Therefore, clearing damaged mitochondria to perform mitochondrial quality control is vital for cellular health (168). Mitophagy, as a key pathway of selective autophagy, achieves mitochondrial quality control by selectively eliminating damaged mitochondria (**Figure 5**). Mitophagy is mainly divided into two categories: one is the ubiquitination pathway, which is mainly the PINK 1-Parkin pathway-mediated mitophagy and receptor-initiated pathway. In the PINK 1 -induced (PTEN induced putative kinase 1) pathway, PINK 1 regulates the active of Parkin during mitochondrial depolarization (169). In the damaged mitochondria, the altered mitochondrial membrane potential and PARL protease activity promote the recruitment of PINK 1 to the outer mitochondrial membrane. PINK 1-mediated phosphorylation transforms Parkin from the inactive form to the active form upon mitochondrial depolarization, Then ubiquitinate some mitochondrial outer membrane proteins, Polyubiquitinated protein is recognized by p62 (p62/Sequestosome-1) and associated with LC3 (microtubule-associated proteins 1A/1B-light chain 3, MAP1LC3) Interactions to form autophagic vesicles to produce phagosomes to remove damaged mitochondrial (170), Mediates defective mitochondria (168). In the non-ubiquitinated receptor initiation pathway, FUNDC1 (FUN14 domain-containing 1) and others act as receptors for hypoxia-induced mitophagy, and dephosphorylation interacts with LC3 to induce mitophagy (171, 172). Under adverse conditions such as hypoxia, stimulation of FUNDC1 receptors activates mitophagy receptors, thereby further promoting the formation of autophagosomes that mediate mitophagy (173). Mitophagy, as a cellular protective mechanism, maintains mitochondrial homeostasis by selectively clearing damaged mitochondria. Its activation is closely linked to mitochondrial functional status. Metabolites released from damaged mitochondria—such as mtROS, ATP, and mtDNA—can trigger mitophagy through ubiquitin-dependent pathways (e.g., the PINK1/Parkin pathway) and ubiquitin-independent (receptor-mediated) pathways (174–176). Mitochondrial dynamics are intimately associated with mitophagy regulation. The mitochondrial fission protein DRP1 significantly influences both Parkin-mediated mitophagy and BNIP3-related mitophagy (177, 178). Overexpression of the mitochondrial fusion protein MFN2 promotes mitophagy via the PINK1/Parkin pathway (179), while MFN2 deficiency can trigger BNIP3-related mitophagy (180).

### 6.3 Mitochondria and selective autophagy in the pathogenesis of OP

As a complex dynamic process, autophagy is widely involved in bone tissue metabolism. An increasing number of studies also demonstrate that mitochondrial autophagy plays a key role in the proliferation and differentiation of bone-related cells, such as osteoblasts, osteoclasts, and mesenchymal stem cells (181). Research indicates that LRRc17 knockdown can inhibit BMSCs senescence by activating mitophagy, thereby enhancing BMSCs’ therapeutic efficacy for osteoporosis (182). Li et al. demonstrate that PINK1/Parkin-mediated mitophagy reduces AOPP-induced osteoblast apoptosis, improving bone loss, microstructural deterioration, and bone mineral density (BMD) impairment (183); Maity et al. find that inducing Kruppel-like factor 2 (KLF2)-mediated mitophagy stimulates DPSCs differentiation toward osteoblasts (184). Conversely, Pink1-/- mice exhibit significantly reduced bone mass and collagen deposition in femurs (184), while Pink1-downregulated MC3T3-E1 cells show impaired mitochondrial homeostasis with suppressed osteogenic differentiation (185). Pink1 silencing or deficiency further promotes excessive osteoclast differentiation and bone resorption (186). Additionally, interventions targeting ROS and mitochondrial dynamics-related proteins influence mitophagy and bone metabolism mechanisms; similarly, L-arginine treatment effectively ameliorates osteoporosis in ovariectomized mouse models; Ling et al. reveal that Sirtuin3 (Sirt3) deficiency induces excessive osteoclast ROS production and autophagy loss, accelerating age-induced bone impairment (187). Another study shows that inducing ROS production and recruiting PINK1/Parkin enhances mitophagy, while activating DRP1 strengthens mitophagy pathways to promote mitochondrial fission and osteoblast differentiation (188). Chen et al. confirm that upregulating the PINK1/DRP1 pathway activates mitophagy to restore osteoblast differentiation capacity (189). MFN2 critically maintains bone homeostasis through mitochondrial network regulation, suppressing oxygen consumption during early osteogenesis to limit differentiation and cortical bone accumulation (123). Reduced MFN2 expression correlates with impaired osteoblast differentiation/function and decreased viable osteocytes (190). Studies demonstrate MFN2 mediates mitochondria-ER tethering to facilitate mitophagy, and its tethering function controls osteoclast differentiation via the Ca²^+^-NFATc1 axis (190). Furthermore, inhibiting mTOR/PI3K signaling promotes mitophagy to prevent BMSCs senescence, whereas mitophagy inhibition accelerates this process (191). These studies provide new therapeutic strategies for osteoporosis (**Supplementary Table 5-1**).

### 6.4 Targeting mitophagy: a therapeutic strategy for bone-related disorders

Targeting mitophagy to regulate bone-related cells may serve as an effective strategy for improving osteoporosis (**Supplementary Table 5-2**). Recent studies have found that enhancing PINK 1 activity, possibly by triggering selective autophagy of mitochondria, and participating in mitochondrial regeneration in improving bone loss in an ovariectomy mouse model (185). Another study found that the HIV proteins Tat and Nef could promote human BMSCs senescence and alter osteoblast differentiation through mitochondria-associated autophagy (192). besides, The study found that 17 β -estradiol could be expressed through GPR 30 (G protein-coupled receptor 30) and ERK 1/2 (Extracellular-regulated kinase 1/2) signaling pathways, Enhanced mitophagy in the mouse MC3T3-E1 osteoblast cell line (193); Small interfering RNAmiR-21-5p can regulate the mitochondria-associated mitochondrial fission and fusion processes, Thus promoting the BMSCs dry recovery of senile OP, enhancing the expression of the osteogenesis-related gene RUNX-2, While reducing of TRAP accumulation in cells with deteriorating phenotypes (194). Metal ion high Mg^2+^ can also regulate MVs mediated mineralization by inhibiting mitochondrial calcium, regulate autophagy of hBMSCs during osteogenesis, leading to reduced deposition of extracellular mineralization matrix, and exogenous ATP can reverse the inhibitory effect of high Mg^2+^ by increasing autophagy levels (195). Upregulation of Parkin and downregulation of P53 in BMSCs could significantly enhance mitophagy in BMSCs and reduce the accumulation of damaged mitochondria in cells, thus effectively resisting stress-induced apoptosis and senescence of BMSCs (196). At present, there are relatively few studies on targeting mitophagy to improve OP, and the specific mechanism is still unclear and needs further study.

## 7 Conclusion

Mitochondria, as organelles with multifaceted biological functions, exhibit dysfunction closely linked to diverse pathophysiological processes (197). Recent research demonstrates their pivotal role in OP pathogenesis through regulating energy metabolism and multiple PCD pathways (10). This review systematically elucidates mitochondrial mechanisms governing bone cell fate via apoptosis, necroptosis, pyroptosis, ferroptosis, and autophagy, revealing intimate connections between mitochondrial dysfunction and OP pathological progression. At molecular level, mitochondria-derived bioactive molecules exert multifaceted regulatory effects on PCD: mtROS serves not only as a trigger for apoptosis and necroptosis (67, 198), but also drives pyroptosis and ferroptosis cascades by activating the NLRP3 inflammasome and promoting lipid peroxidation (135, 146, 147). Additionally, abnormal mtDNA release has been confirmed to modulate multiple PCD pathways including apoptosis (18), necroptosis (70), and pyroptosis (132).

Notably, significant crosstalk exists among multiple programmed cell death (PCD) pathways in osteoporosis; within mitochondrial regulation of OP-related PCD, mitochondria coordinate apoptosis through Bcl-2 family proteins and necroptosis via RIPK1/3 signaling, with these two pathways maintaining dynamic equilibrium that collectively impacts bone homeostasis (69). Mitophagy not only suppresses pyroptosis in BMDM cells but also inhibits ferroptosis in osteoblasts (130, 157). These findings indicate that mitochondrial dual-regulation of apoptosis/necroptosis balance and the pleiotropic effects of mitophagy represent crucial future research directions for OP.

Despite the promising therapeutic potential of mitochondrial targeting in OP, several key scientific questions require resolution: First, the precise regulatory networks through which mitochondria coordinate different PCD pathways need further elucidation; second, the heterogeneity of mitochondrial functions among distinct bone cells—including osteoblasts, osteoclasts, and osteocytes—and its pathological significance demand in-depth investigation.

Collectively, the multidimensional regulatory properties of mitochondria in OP provide novel perspectives for understanding bone metabolism complexity; future research should integrate cutting-edge technologies like single-cell sequencing and gene editing to target key mitochondrial regulatory nodes, thereby advancing the development of novel anti-osteoporosis therapeutics with enhanced theoretical foundations and druggable targets.

All images were created using BioRender (https://biorender.com/).
