# Supplementary material for: Mitochondria: a key regulator of programmed cell death in OP
Source: Front Endocrinol (Lausanne). 2025 Jul 2;16:1576597. doi: 10.3389/fendo.2025.1576597 (PMC12263366; doi:10.3389/fendo.2025.1576597)
Supplement: Supplementary file 1 [file DataSheet1.docx]

**Tab. 1-1 Mitochondria-Mediated Apoptosis in the Pathogenesis of OP**

| **Diseases** | **Cells processing** | **The cells used** | **Animals handling** | **Animals used** | **Effects on mitochondria** | **Effects on bone/bone-associated cells** |
| --- | --- | --- | --- | --- | --- | --- |
| Osteoporosis due to zinc deficiency | N,N,N,′,N′-tetrakis (2-pyridylmethyl) ethylenediamine , Zinc | MC3T3-E1 |  |  | Translocate Bax into mitochondria, reduce mitochondrial membrane potential, and promote the release of Cyt c and ATF from mitochondria | Induction of apoptosis through a mitochondria-mediated pathway |
| Glucocorticoid-induced osteoporosis | β-glycerol phosphate, L-ascorbic acid, mRANKL,  Dexamethasone | MC3T3-E1、RAW264.7 |  |  | resulting in altered mitochondrial integrity and function and Cyt c release in osteoblasts | Induction of apoptosis in osteoblasts |
| Glucocorticoid-induced osteoporosis | β-glycerol phosphate, L-ascorbic acid, Dexamethasone | MC3T3-E1 |  |  | Early increases in mitochondrial activity and biogenesis, followed by mitochondrial fission. | Induction of apoptosis |
| Osteoporosis due to iron overload | Ferric ammonium citrate , N-acetyl-cysteine, Osteogenesis differentiation media | MC3T3-E1, BMSCs | Isolate BMSCs | Sprague-Dawley rats | affecting mitochondrial membrane potential, mitochondrial depolarization and release of mitochondrial cytochrome c | Affecting cellular ROS levels and thus apoptosis |
| Osteoporosis secondary to diabetes |  |  |  |  | Mitochondrial DNA point mutations and mitochondrial dysfunction | Associated with reduced bone mineral density, cortical thinning, and diminished bone strength |
| Diabetes-related osteoporosis |  |  |  |  | Mitochondrial DNA point mutations | Mitochondrial DNA point mutations had the highest rate of osteoporosis |
| Osteoporosis due to radiotherapy |  | Bone marrow cells | Radiation, 1,4-Diamino-2,3-dicyano-1,4-bis (o-aminophenylmercapto) butadiene | Mice | The mitochondrial transmembrane protein OPA, enhanced mitochondrial ATP | Inhibition of apoptosis of bone marrow cells |

**Abbreviations:** Apoptosis-inducing factor (AIF); Cytochrome c (Cyt c); Mouse embryo osteoblast precursor cells (MC3T3-E1); membrane-bound Receptor Activator of Nuclear Factor Kappa-B Ligand (mRANKL); Mouse monocytic macrophage leukemia cell line (RAW264.7); Bone marrow-derived mesenchymal stem cells (BMSCs); Reactive oxygen species (ROS); A to G transition at position 3243 of mitochondrial DNA (m.3243A > G).
