# Supplementary material for: Mitochondria: a key regulator of programmed cell death in OP
Source: Front Endocrinol (Lausanne). 2025 Jul 2;16:1576597. doi: 10.3389/fendo.2025.1576597 (PMC12263366; doi:10.3389/fendo.2025.1576597)
Supplement: Supplementary file 2 [file DataSheet2.docx]

**Tab.1-2 Targeting Mitochondrial Apoptosis: A Therapeutic Strategy for Bone-Related Disorders**

| **Diseases** | **Cells processing** | **The cells used** | **Animals handling** | **Animals used** | **Type of drugs** | **Drugs** | **Improving the mitochondrial pathway** | **Effects on mitochondria** | **Effects on bone/bone-associated cells** |
| --- | --- | --- | --- | --- | --- | --- | --- | --- | --- |
| Osteoporosis | H_2_O_2,_ Metformin, SIRT3-siRNA | MC3T3-E1 | Ovariectomy, Metformin | C57BL/6J mice | Common clinical drugs | Metformin | Upregulation of SIRT3 expression through the PI3K/AKT pathway | Improvement of mitochondrial membrane potential, Repair of mitochondrial damage | Reversal of osteoblast apoptosis |
| Postmenopausal osteoporosis | M-CSF, RANKL, R&D Systems Minneapolis, E_2_ | BMMs | Ovariectomy, Isolate BMMs, E_2_, Rotenone | FasL^gld/gld^ mice,C57BL/6J(B6) mice, ERα^ΔLysM^ mice, ERα^f/f^ mice, ERα^f/+^ mice, Bak^ΔBaxf/f^ mice, LysM-Cre mice, Bak^ΔBax+/f^ mice, Bak^ΔLysM-Cre^ mice, Bak^ΔBaxf/f^ mice, Bak^+/−^ mice, Bak^ΔBaxΔLysM^ mice | Common clinical drugs | E_2_ |  | Inhibition of mitochondrial genes and mitochondrial respiration in osteoblast progenitor cells | Promoting apoptosis of early osteoclast progenitors |
| Postmenopausal osteoporosis | Serum starvation, Naringin | VECs | Naringin, Ovariectomy | Sprague-Dawley mouse | Herbal monomers | Naringin |  | Inhibition of Cyt.c protein release into the cytoplasm, restoration of mitochondrial membrane potential, the | Inhibition of mitochondria-mediated apoptosis of VEC cells, increase in bone density, the |
| Osteoporosis | RANKL, Naringin | RAW264.7 | Naringin, Ovariectomy, 17-b estradiol, tetracycline | Sprague-Dawley mouse | Herbal monomers | Naringin |  | Reduced levels of Cyt.c | By regulating the activity of the mitochondrial apoptosis pathway, it promotes osteoclast apoptosis and prevents OVX-induced osteoporosis in rats |
| Osteoporosis | RANKL, Vanillin, M-CSF | RAW264.7 |  |  | Herbal monomers | Vanillin |  | Induction of Cyt.c release | Activation of mitochondria-dependent osteoclast apoptosis attenuating the bone resorption capacity of osteoclasts |
| Inflammatory bone destruction and postmenopausal osteoporosis | MCS-F, RANKL, Euphorbia factor L1 | BMMs | Ovariectomy, Euphorbia factor L1, LPS | C57BL/6 mice | Herbal monomers | Euphorbia factor L1 | Inhibition of mRNA expression of PGC-1β and its target genes (including ND4, COX1, COX3), which regulate mitochondrial biogenesis, and protein expression of PGC-β1, the | Enhancing mitochondrial biogenesis | Inhibits osteoclast formation and function and promotes osteoclast apoptosis during the initial and late stages of osteoclast formation; Inhibition of bone loss in ovariectomized mice |
| Osteoporosis | H_2_O_2_、  GSK-3β Inhibitor I (TDZD-8), N-Acetylcysteine, A potent and specific cell-permeable inhibitor of phosphatidylinositol 3-kinase (LY294002), Hydroxytyrosol (3,4-dihydroxyphenylethanol (HT)), OPAI-siRNA, Control-siRNA | MC3T3-E1 |  |  | Herbal monomers | Hydroxytyrosol (3,4-dihydroxyphenylethanol (HT)) | blocking OPA1 cleavage and increasing Akt-GSK3β signaling | Improved mitochondrial dysfunction (mtROS, MMP, ATP), Conservation of mitochondrial morphology | Preventing osteoblast apoptosis through an OPA1-dependent mitochondrial pathway |
| Glucocorticoid -induced osteoporosis | Puerarin, Glucocorticoid, Dexamethasone, E_2,_ Anthrapyrazolone inhibitor of Jun N-terminal kinase , LY294002, Fulvestrant | hFOB19.3 |  |  | Herbal monomers | Puerarin | Inhibition of the JNK pathway and activation in PI3K/Akt signaling pathways | Attenuated dexamethasone-induced cytochrome c release | Regulation of apoptosis in osteoblasts |
| Glucocorticoid -induced osteoporosis | Dexamethasone, Sulforaphane, Alpha minimum essential medium, z-VAD-FMK, Caspase-3 Inhibitor (Z-DEVD-FMK), Caspase-9 Inhibitor (Z-LEHD-FMK), Caspase-8 Inhibitor (Z-IETD-FMK), Nrf2-siRNA | MC3T3-E1 |  |  | Herbal monomers | Sulforaphane | Nrf2 pathway | Inhibition of the mitochondria-mediated apoptosis pathway that | Reversal of mitochondria-mediated apoptosis in osteoblasts |
| Glucocorticoid -induced osteoporosis | Tanshinone IIA, Dexamethasone, Z-LEHD-FMK, Z-IETD-FMK, Z-VAD-FMK, N-Acetylcysteine, NADPH oxidases inhibitor diphenyleneiodonium (DPI), Apocynin, Rotenone, Allopurinol,Plumbagin, Nox4-siRNA, Negative | MC3T3-E1 |  |  | Herbal monomers | Tanshinone IIA | Inhibition of Nox4 expression | Inhibition of cytochrome c release from mitochondria | Inhibition of mitochondria-mediated apoptosis in osteoblasts |
| Osteoporosis | Grape seed proanthocyanidins, H_2_O_2_ | MC3T3-E1 |  |  | Herbal monomers | Grape seed proanthocyanidins | Promoting the restoration of mitochondrial electron transport chain function | Reduces mitochondrial free radical production, ameliorating mitochondrial dysfunction | Inhibition of apoptotic signaling in osteoblasts |
| Osteoporosis | Curcumin, H_2_O_2_, Ethylbisminomethylguaiacol manganese chloride (EUK134), TDZD-8、LY294002 | Saos-2 |  |  | Herbal monomers | Curcumin | Increased Akt-GSK3β signaling | Improved mitochondria oxidative state, potentiation, and improved mitochondrial function | Attenuating oxidative stress-induced apoptosis in osteoblasts |
| Osteoporosis secondary to diabetes | Advanced glycation end products ,MitoQ Mitoquinone, CsA, Silibinin, RAGE Antagonist (FPS-ZM1), RAGE, Bovine Serum Albumin, H_2_O_2_ | MC3T3-E1 |  |  | Herbal monomers | Silibinin | down-regulates the expression of RAGE | mtROS accumulation, mitochondrial membrane potential disruption, morphological alterations, dysregulated dynamics, and impaired ATP production | Inhibition of AGE-induced apoptosis in osteoblasts |
|  | Crocin, Dexamethasone, N-Acetylcysteine, BAP | MC3T3-E1 |  |  | Herbal monomers | Crocin | Increased levels of ROS/Ca²+ | Mitigating mitochondrial dysfunction (restoration of mitochondrial membrane potential; inhibition of cytochrome c release from mitochondria) | Inhibition of the mitochondrial apoptotic pathway in osteoblasts |
| Osteoporosis | H_2_O_2,_ SP600125, Anisomycin, Pre‐incubated、Osteogenic medium , β‐glycerol phosphate, Ascorbic acid, Notoginsenoside R1 | MC3T3-E1 |  |  | Herbal monomers | Notoginsenoside R1 | Blocking the JNK signaling pathway | Mitigating mitochondrial dysfunction (restored MMP, improved mtROS production, increased ATP production, and MtDNA copy number) | Ameliorating osteoblast apoptosis and dysfunction |
| Osteoporosis secondary to cancer chemotherapy | Cisplatin, Spirulina Platensis protein, Se-containing S. platensis protein, H_2_O_2,_ CsA, GSH | MC3T3-E1 |  |  | Herbal monomers | Se-containing S. platensis protein |  | Restoring mitochondrial dysfunction (regulates mitochondrial membrane potential) | Inhibition of mitochondria-mediated apoptosis and oxidative damage |
| Osteoporosis | H_2_O_2_, Fermented oyster extracts, N-Acetylcysteine, Nrf2-siRNA, Control-siRNA | MC3T3-E1 |  |  | Herbal monomers | Fermented oyster extracts | activated the Nrf2/HO-1 signaling pathway | Mitigating mitochondrial dysfunction (restored MMP, inhibited cytochrome c release from mitochondria) | Inhibition of mitochondria-associated apoptosis |
| Osteoporosis | H_2_O_2,_ ,Simvastatin | MG63 cells(human osteosarcoma cell) |  |  | Clinical Drugs | Simvastatin |  | Activation of caspase-9, the most upstream protease in the mitochondrial apoptotic pathway | Inhibition of oxidative Stress-induced apoptosis through mitochondria-mediated signaling |
| Osteoporosis | Astragalus polysaccharide , Ferri Ammonii Citras | BMSCs | Isolate BMSCs cells | C57BL/6J mice | Herbal monomers | Astragalus polysaccharide |  | Inhibition of mitochondrial superoxide production | Significantly inhibited Ferri Ammonii Citras-induced apoptosis, proliferation and pluripotency of BMSC induced by iron overload |
| Osteoporosis,Bone loss | M-CSF, RANKL, β-glycerophosphate, L-ascorbic acid, dexamethasone, P. mirabilis OMVs, Escherichia coli OMVs, Lactobacillus casei OMVs, Lactobacillus acidophilus OMVs, P. mirabilis, LPS | BMMs、MC3T3-E1 | Ovariectomy, Collagen-Induced Arthritis , PBS, Proteus mirabilis OMVs, Escherichia coli OMVs, miR-96-5p mimic, miR-96-5p inhibitor | DBA/1 mice、C57BL/6J mice | Other | Proteus mirabilis OMVs | Inhibition of miR96-5p | Elevation of caspase-3 expression and mitochondrial ROS levels, promotion of cytochrome c release from mitochondria | Promoting mitochondria-dependent apoptosis in osteoclasts and alleviating bone flow patterns that |
| Osteoporosis secondary to diabetes | Normal glucose、High glucose, Glycyrrhizin, Dorsomorphin 2HCl, Osteogenic cultural medium | BMSCs | Isolate BMSCs cells | Sprague-Dawley rats | Herbal monomers | Glycyrrhizin | Inhibition of AMPK activation by HMGB1 | Alleviating mitochondrial dysfunction and restoring mitochondrial morphology | Alleviating high glucose-induced apoptosis in BMSCs |
| Hormone-related secondary osteoporosis | H_2_O_2_, An analogue of Humanin (HNGF6A), pLV5-Circ_0001843,miR-214 inhibitor, Adezmapimod (SB 203580) , JNK1/2/3 inhibitor (SP600125) | MC3T3-E1 |  |  | Other | HNGF6A |  | HNGF6A is an analogue of mitochondrial derived peptide | Protection of osteoblasts against oxidative stress-induced apoptosis and restoration of osteogenic differentiation capacity of osteoblasts |

**Abbreviations:** Hydrogen peroxide (H_2_O_2_); Sirtuin 3 (SIRT3); Small-interfering RNA (siRNA); Macrophage colony stimulating factor (M-CSF); Receptor activator for nuclear factor-κB ligand (RANKL); Bone marrow-derived macrophages (BMMs); Vascular endothelial cells (VECs); Human Fetal Osteoblastic Cell Line 1.19 (hFOB1.19); Estrogen (E_2_); Phosphatidylinositol-3-kinase (PI3K); Protein kinase B (Akt); Factor-related Apoptosis ligand (FASL); Estrogen Receptor alpha（ERα）; BCL2 associated X, apoptosis regulator (Bax) ; BCL2 antagonist/killer 1 (Bak); Myeloid cell-specific gene (LysM); Cyclization Recombination Enzyme (Cre); Mitochondrial ROS (mtROS); Optic atrophy 1 (OPA1); Mitochondrial membrane potential (MMP); Glycogen synthase kinase 3β (GSK3β); Jun N-terminal kinase (JNK); Nuclear factor erythroid 2-related factor 2 (Nrf2); Heme oxygenase-1 (HO-1); Benzyloxycarbonyl-Val-Ala-Asp (OMe)-fluoromethylketone (Z-VAD-FMK); NADPH Oxidase 4 (Nox4); Osteoblastic cell line (Saos-2); Cyclosporin A (CsA); Receptor of AGEs (RAGE); Glutathione (GSH); Phosphate buffered saline (PBS); High mobility group box protein 1 (HMGB1); Lipopolysaccharide (LPS); Outer membrane vesicles (OMVs); Mitochondrial ferritin (FtMt)；Lentiviral vector ( LV); Adenosine 5'-monophosphate (AMP)-activated protein kinase (AMPK); Reactive Oxygen Species Inhibitor(HNGF6A)
