# Supplementary material for: Mitochondria: a key regulator of programmed cell death in OP
Source: Front Endocrinol (Lausanne). 2025 Jul 2;16:1576597. doi: 10.3389/fendo.2025.1576597 (PMC12263366; doi:10.3389/fendo.2025.1576597)
Supplement: Supplementary file 3 [file DataSheet3.docx]

**Tab.2-1 Mitochondria-Driven Necroptosis as a Key Pathway in the Development of OP**

| **Diseases** | **Cells processing** | **The cells used** | **Animals handling** | **Animals used** | **Effects on mitochondria** | **Effects on bone/bone-associated cells** |
| --- | --- | --- | --- | --- | --- | --- |
| Postmenopausal osteoporosis | Nec-1, zVAD, TNF-α, DMSO | MLO-Y4 | Ovariectomy, Sham operation, Nec-1, zVAD | Sprague Dawley rats |  | Combined blockade of osteoclast necroptosis and apoptosis reduces osteoclast loss and alleviates bone flow patterns |
| Postmenopausal osteoporosis | Nec-1, TNF-α | MLO-Y4 | Ovariectomy, sham operation, Nec-1, zVAD, E_2_ | Sprague Dawley rats |  | An inhibitor of necrotic apoptosis prevents E_2_ deficiency-induced osteoclast necrosis in Ovariectomy rats and attenuates bone flow patterns |
| Bone loss | BaP, Nec-1, SP600125, N-Acetylcysteine | MLO-Y4 | BaP, Corn oil | BALB/c mice | Affecting the generation of mtROS | BaP promotes necrotic apoptosis induced by oxidative stress in osteoblasts in vitro and accelerates bone loss |
|  | Ferric ammonium citrate , N-Acetylcysteine, Nec-1, Necrosulfonamide , RIPK3 inhibitor( GSK872), RIPK1-siRNA、RIPK3-siRNA | MC3T3-E1 |  |  | Generation of mtROS, opening of mPTP, and loss of mitochondrial membrane potential | Affecting iron overload-induced necrotic apoptosis in osteoblasts |
| Glucocorticoid-induced osteoporosis | Dex, z-VAD-fmk, Nec-1 | MC3T3-E1 |  |  | Causing mitochondrial swelling, altering mitochondrial membrane potential and ATP levels, and | Necroptosis and apoptosis of osteoblasts accompanied by alterations in mitochondrial function and morphology |
| The dysregulation of bone marrow microenvironment | RIPK1-siRNA, p53-siRNA, Control-siRNA | MSCs | Isolate MSCs | Sprague Dawley rats | Affecting mitochondrial homeostasis | Reduced the proliferative differentiation capacity of BMSC, induced apoptosis and necrotic apoptosis |
| Diabetic kidney disease | D-glucose, High glucose | MSCs, GECs | STZ, HBSS, MSCs | Sprague Dawley rats | Diverted mitochondria, affecting mitochondrial dynamics (mitochondrial ROS generation, levels of mitochondrial fusion and fission factors) | Significantly altered apoptosis and viability |
|  |  | RAW264.7 | Cigarette smoke extract |  | A decrease in mitochondrial membrane potential, an increase in ROS production, and a decrease in ATP concentration that | Resulting in decreased cell activity and necrosis |
| Cancer | Cisplatin, A. muricata leaf polysaccharides , Macrophage colony-stimulating factor, Cisplatin | RAW264.7, BMDMs, Human lung cancer cell lines (A549 and H460) | Isolate BMDMs | C57BL/6 mice | Affecting reactive oxygen species production and mitochondrial transmembrane potential | Altering cellular damage by affecting the mitochondrial apoptotic pathway |
| Intervertebral disc degeneration | LPS, Z-VAD, N-acetylcysteine, Blebbistatin, Y27632, Staurosporine | nucleus pulposus (NP) cells |  |  | NP cells released by cells contain a mitochondrial component, and the cell-released mitochondria are granular in nature, the | NP cells containing mitochondria can induce further cell death |

**Abbreviations:** Pan-Caspase Inhibitor (zVAD); Tumor necrosis factor-α (TNF-α); Necrostatin-1 (Nec-1); Murine long bone osteocyte Y4 (MLO-Y4); Business approval process (Bap); Receptor-interacting protein kinase 1 (RIPK1); Receptor-interacting protein kinase 13(RIPK3); Cellular tumor antigen p53 (p53); Mesenchymal stem cells (MSCs); Glomerular Endothelial Cells (GECs); ; Rho-associated kinase inhibitor (Y27632); Macrophages (MPs)
