# Supplementary material for: Mitochondria: a key regulator of programmed cell death in OP
Source: Front Endocrinol (Lausanne). 2025 Jul 2;16:1576597. doi: 10.3389/fendo.2025.1576597 (PMC12263366; doi:10.3389/fendo.2025.1576597)
Supplement: Supplementary file 4 [file DataSheet4.docx]

**Tab.2-2 Inhibiting Mitochondrial Necroptosis: A Therapeutic Strategy for Bone-Related Diseases**

| **Diseases** | **Cells processing** | **The cells used** | **Animals handling** | **Animals used** | **Type of drugs** | **Drugs** | **Improving the mitochondrial pathway** | **Effects on mitochondria** | **Effects on bone/bone-associated cells** |
| --- | --- | --- | --- | --- | --- | --- | --- | --- | --- |
| The dysregulation of bone marrow microenvironment | RIPK1-siRNA、p53-siRNA、Control-siRNA | MSCs | Isolate MSCs | Sprague Dawley rats | Targeted inhibition | Knock down p53 | p53-PUMA shaft | Affecting mitochondrial homeostasis | Affects the ability of BMSCs to proliferate and differentiate, regulates apoptosis and necrotic apoptosis, and |
|  |  |  |  |  |  |  |  |  |  |
| Intervertebral disc degeneration | TNFα, LPS, z-VAD, MyD88, Inhibitory peptide | NP cells | Isolate NP cells | Sprague Dawley rats | Targeted inhibition | MyD88 inhibitor | MyD88 pathway | Protecting mitochondria | Inhibition of necrotic apoptosis in NP cells, the |
| Intervertebral disc degeneration | Amo, Tert-butyl hydrogen peroxide, N-Acetylcysteine, SP600125 | NP cells | Isolate NP cells | New Zealand White rabbit | Common clinical drugs | Amo | Mitogen-activated MAPK signaling pathway | Protecting mitochondrial function and reducing mitochondrial levels of oxidative stress | Reduced tBHP-induced necrotic apoptosis and ROS production |
| Intervertebral disc degeneration | Compression, Nec-1, Z-VAD-FMK, N-Acetylcysteine, BHA, mPTP Inhibitor CsA, DMSO, RIPK1-siRNA | NP cells | Isolate NP cells | Sprague Dawley rats | Inhibitors | Nec-1， .CsA | RIPK1 | Restoration of mitochondrial ultrastructure and amelioration of mitochondrial dysfunction | Ameliorating the oxidative stress state of NP cells, inhibiting apoptosis in the mitochondrial pathway, and further promoting necrotic apoptosis |
| Intervertebral disc degeneration | Compression, Nec-1, 3-Methyladenine, Z-VAD-FMK | NP cells | Isolate NP cells | Sprague Dawley rats | Inhibitors | Necrostatin-1, Z-VAD |  | Improvement of mitochondrial dysfunction (MMP, MPTP and oxidative stress index) | Jointly ameliorating oxidative stress in cells and inhibiting cell death, the |
| Osteoarthritis | IL-1β, TNF-α, Insulin–transferrin–selenium-A–DMEM, Mitochondria、Co-culture | Chondrocytes, L6 cells | Monosodium iodoacetate , mitochondria | Wistar rats |  | Mitochondrial transplantation |  | Improving mitochondrial function (mitochondrial membrane potential, mitochondrial swelling, cristae structural changes, mitochondrial number, triggered autophagy) | Decreased markers of necrotic apoptosis, the reducing cartilage destruction and bone loss in the knee joint |
| Spinal cord injury |  |  | Laminectomy, spinal cord injury, Nec-1 | Sprague Dawley rats | Inhibitors | Necrostatin-1 (Nec-1) |  | Protecting the structure and function of mitochondria | Attenuating necrotic apoptosis and promoting cytoprotection and physiological function after spinal cord injury |
| Ischemia-reperfusion episodes | M-CSF, LPS, IL-4, PMA, The Control cell line (pCMV3-EMPTY), pCMV3-PARP1, H_2_O_2_, PARP1 inhibitor (PJ34) | BMDMs, THP-1 | PARP1 KO | Mice |  | LPS |  | Affecting the expression of the mitochondrial enzyme SOD2, affecting mitochondrial respiration | Affect PARP1-mediated cell death |

**Abbreviations:** Myeloid differentiation primary response protein 88 (MyD88); Amobarbital (Amo); Butylated hydroxyanisole (BHA); Mitochondrial permeability transition pore (mPTP); Interleukin—1β(IL-1β); L-6 myoblast cells line (L6 cells); Interleukin 4 (IL-4); Phorbol 12-myristate 13-acetate (PMA); Knockout (KO); Plasmid Cytomegalovirus Promoter Vector 3(pCMV3); Poly (ADP-ribose) polymerase-1 (PARP1); Superoxide dismutase 2 Gene (SOD2)
