# Supplementary material for: Mitochondria: a key regulator of programmed cell death in OP
Source: Front Endocrinol (Lausanne). 2025 Jul 2;16:1576597. doi: 10.3389/fendo.2025.1576597 (PMC12263366; doi:10.3389/fendo.2025.1576597)
Supplement: Supplementary file 5 [file DataSheet5.docx]

**Tab.3-1 Mitochondria-Regulated Pyroptosis in the Pathogenesis of OP**

| **Diseases** | **Cells processing** | **The cells used** | **Animals handling** | **Animals used** | **Effects on mitochondria** | **Effects on bone/bone-associated cells** |
| --- | --- | --- | --- | --- | --- | --- |
| Postmenopausal osteoporosis | De-ovulatory,  Dexamethasone, Ascorbic acid, Glycerophosphate | BMSCs | NLRP3 knockout, Ovariectomy | C57BL/6J mice |  | High levels of inflammation induced by NLRP3-induced cellular pyroptosis inhibited osteogenic differentiation of osteoblasts. |
|  | H_2_O_2_, siUSP1, oeUSP1 | MC3T3-E1 |  |  |  | USP1 can regulate NF-κB signaling by stabilizing TRAF6 to influence cellular focal death |
| Periodontitis | LPS, shRNA NC, TRPM2 shRNA, NLRP3 shRNA, Osteogenic induction media, Chondrogenic induction media, Adipogenic induction media | BMSCs | Post-Periodontitis Tooth Extraction | TRPM2-/- [TRPM2 knockout (KO)] mice, C57BL/6 mice | Increased mitochondrial dynamics | Inhibition of NLRP3 inflammatory vesicle activation regulates cellular pyroptosis and mitochondrial dynamics-mediated osteogenesis |
| Osteoporosis | RANKL, M-CSF, L-ascorbic acid, β-glycerophosphate, LPS, Opti-MEM, Nigericin, ATP, Nec-1, A small molecule caspase-1 inhibitor (VX-765), Z-DEVD-FMK, Z-IETD-FMK, Z-VAD-FMK, A novel PIKfyve-specific inhibitor (YM201636) | BMDMs, HEK293T and HeLa cells | Isolate BMDMs, Ovariectomy, Induce aging | C57BL/6J mice, Gsdmd-/- mice, Lyz2-Cre mice, Gsdmd FL/FL mice, Nlrp3-/- mice, Casp1-/- mice, Asc-/- mice, Casp11-/- mice, LC3-GFP mice |  | GSDMD inhibits osteoclast bone resorption activity, the |
| Atherosclerosis | ox-LDL, sh-H19, Lv-H19, VX-765, MnTBAP | Raw 264.7 |  |  | Inhibiting mitochondrial dysfunction | Inhibited intracellular ROS accumulation and induced induced endothelial cell pyroptosis, the |
| Inflammatory diseases | LPS , PMA, Apilimod, Selective and direct NLRP3 inhibitors (CY-09), Potent and selective NLRP3 inhibitor (MCC950), Chimeric triple action NLRP3 inhibitor (RRx-001), Nigericin, GFP-NLRP3, Rab7 receptor antagonist (CID1067700), Bafilomycin A, Specific Cathepsin B inhibitor (CA-074-Me), Membrane-permeable Ca^2+^ chelator (BAPTA-AM), 2-APB, G6pdx-siRNA, Trpml1-siRNA, Ctsb-siRNA | BMDMs, THP-1, PBMCs, Hela cells | Isolate BMDMs | C57BL/6J mice, Gsdmd^-/-^ mice, Nlrp3^-/-^ mice, Aim2^-/-^ mice, ASC^-/-^ mice, Caspase1^-/-^ mice, Mevf^-/-^ mice | Inducing mitochondrial damage and ROS production | Activation of NLRP3 inflammasome and promotion of cellular pyroptosis in a mitochondria-dependent manner |
| Inflammatory diseases | LPS, nigericin, AMA, TcdB, glycine, punicalagin, staurosporin, ATP | BMDMs, HEK293T | Isolate BMDMs | C57BL/6, P2rx7-/- mice, Nlrp3-/- mice, Casp1/11-/- mice, Gsdmd //- mice | Induced mitochondrial damage, resulting in the release of mtDNA from the cell that | Affecting the processes of cellular pyroptosis and apoptosis |
| BPA-induced damage of bone tissue | Bisphenol A , z-VAD-FMK, VX765, N-Acetylcysteine, Mito-TEMPO | MLO-Y4 |  |  | Stimulating cellular mtROS production, the Mitochondrial antioxidants inhibit BPA-induced NLPR3 inflammatory vesicle activation and focal death | Osteoblast pyroptosis via the mitochondria-associated ROS/NLRP3/Caspase-1 pathway |
| Obesity | Insulin, Dexamethasone, 3-isobutyl-1-methyl-xanthine, Rosiglitazone, Atglistatin, TNFα, BMP4, Selective inhibitor of p38 MAPK (SB203580) | SVF, Previpocytes | Isolation of SVF and adipocytes from adipose tissue | FABP4-Cre-BMP4^LoxP/LoxP^ mice, BMPR2^LoxP/LoxP^ mice | Affecting mitochondrial respiratory capacity, expression of mitochondria-associated caspase 9, and release of cytochrome C from within mitochondria | Modulation of functional integrity and survival of adipocytes in inflammatory environments by influencing the mitochondrial pathway to induce apoptosis and pyroptosis |
| Intervertebral disc degeneration | H_2_O_2_ ,Inhibitor of Toll-like receptor (TLR) 7 and 9 (E6446), CsA, TLR9-siRNA | NP cells |  |  | Affecting mPTP opening and cytoplasmic mtDNA release, increased mitochondrial damage, increased | Inhibition of the expression of key proteins of the TLR9-NF-κB-NLRP3 axis regulates NPC focal death, the |
| Intervertebral disc degeneration | MFN2-siRNA (knockdown, overexpression), MSU, NLPR3-siRNA | NP cells |  |  | Mitochondria-related genes-MFN2 were significantly increased in Intervertebral disc degeneration NP tissues | Promotion of NLPR3 expression, activation of NLRP3 inflammatory vesicles, leading to NP cell pyroptosis and release of inflammatory factors |

**Abbreviations:** Nod-like receptor protein 3 (NLRP3); Ubiquitin-specific protease 1 (USP1); Overexpression (oe); Tumor necrosis factor receptor (TNFR)-associated factor 6 (TRAF6); Transient receptor potential melastatin 2 (TRPM2); Short hairpin RNA negative control (shRNA NC) ;Opti-MEM: A serum-reduced media used during transfection of genetic molecules into mammalian cells; HEK293T (human embryonic kidney 293 T antigen-expressing); Short hairpin RNA (shRNA); Oxidized Low-density Lipoprotein (ox-LDL); H19 Imprinted Maternally Expressed Transcript (H19); Bone marrow-derived macrophages (BMDMs); Manganese (III) tetrakis (4-benzoic acid) porphyrin (MnTBAP); Green fluorescent protein (GFP); Phorbol 12-myristate 13-acetate (PMA); 2-Aminoethyl diphenylborinate (2-APB); Glucose-6-phosphate dehydrogenase X-chromosome linked (G6pdx); Transient receptor potential mucolipin 1 (Trpml 1); Cathepsin B (Ctsb); THP-1：Human Acute Monocytic Leukemia Cells； Gasdermin D (Gsdmd);Absent In Melanoma 2 (Aim2); Peripheral blood monoculear cells (PBMCs); Apoptosis-associated speck-like protein containing a CARD (ASC); Murine erythroleukemia virus factor (Mevf); Bone Morphogenetic Protein 4 (BMP4); ; Fatty acid-binding protein 4 (FABP4); Stromal vascular fraction (SVF); BMP Type II Receptor (BMPR2); Toll-like receptor 9 (TLR9); Mitofusin 2(MFN2); Monosodium urate (MSU).
