# Supplementary material for: Mitochondria: a key regulator of programmed cell death in OP
Source: Front Endocrinol (Lausanne). 2025 Jul 2;16:1576597. doi: 10.3389/fendo.2025.1576597 (PMC12263366; doi:10.3389/fendo.2025.1576597)
Supplement: Supplementary file 6 [file DataSheet6.docx]

**Tab.3-2 Targeting Mitochondria to Suppress Pyroptosis: A Therapeutic Strategy for Bone Disorders**

| **Diseases** | **Cells processing** | **The cells used** | **Animal handling** | **Animals used** | **Type of drugs** | **Drugs** | **Improving the mitochondrial pathway** | **Effects on mitochondria** | **Effects on bone/bone-associated cells** |
| --- | --- | --- | --- | --- | --- | --- | --- | --- | --- |
| Pulpitis | LPS, ATP, MitoTEMPO, N-Acetylcysteine, cytochalasin B, NF‐κB inhibitor (Bay 11-7082), TNF-α, IKKβ inhibitor (ML120B), Ethidium bromide, Pyruvic acid, Uridine | mDPC6T, BMSCs |  |  | Mitochondrial transfer | Mitochondria | TNF-α activates the NF-κB signalling promotes mitochondrial transfer | Restoring mitochondrial function and improving mitochondrial redox homeostasis | Reducing NLRP3 inflammatory vesicle-induced odontoblast pyroptosis |
| Myelodysplastic Syndromes | ox-mtDNA, MCC950, NLRP3 KO , TLR9 KO, cGAS OE, RU.521, IRAKi, ODN-F, HCQ, UR521 | THP1, U937, SKM1, BM-MNCs, HEL 92.1.7 |  |  | Inhibitors | Interleukin-1 Receptor-Associated Kinase inhibitor (IRAKi), ODN-F, RU.521 |  | promoting the release of ox-mtDNA from pyroptotic cells | MDS HSPC induces the activation of inflammatory vesicles through the release of ox-mtDNA, leading to a feed-forward loop of cellular pyroptosis, the |
| Spinal cord injury | EPT, Oxygen-glucose deprivation | Primary neurons | Laminectomy, EPT, Oxygen–glucose deprivation，Spinal cord injury | C57BL/6 mice | Mitochondria-targeting peptides | Elamipretide |  | alleviating mt-ROS and mitochondrial dysfunction in primary cultured neurons | Inhibition of pyroptosis and attenuation of inflammation in primary cultured neurons |
| Intervertebral disc degeneration | LPS, SS-31, N-Acetylcysteine | Nucleus pulposus cell |  |  | Mitochondria-targeting peptides | SS-31 |  | limiting mitochondrial ROS production and maintaining mitochondrial dynamic homeostasis | Inhibition of LPS-induced activation of NLRP3 inflammatory vesicles |
| Multiple myeloma | CRISPR-Cas9 KO, Protease inhibitors (PIs), Proteasome inhibitors (MG132), BZ, Nigercin, LPS, pWPI-GSDME, pWPI-GSDME-D270A , N-GSDME-T6E, GSDME KO, GSDME, N-GSDME | Multiple Myeloma cells |  |  | Proteasomal inhibitors | Proteasomal inhibitors | Associated with the interaction of Bcl-2 and Bax | Affecting mitochondrial membrane integrity and promoting Cyto c release | Induction of myeloma cell pyroptosis |
| Osteosarcoma | DCA, OPDEA, OPDEA-PDCA | MNNG-HOS, MG63 and K7M2 cell lines. | OPDEA-PDCA, PEG-DCA, OPDEA, K7M2 tumors were inoculated subcutaneously, Anti-PD-L1 | BALB/C mice | Mitochondria-targeting polymer micelle | Poly[2-(N-oxide-N,N-diethylamino)ethyl methacrylate] |  | Activation of mitochondrial oxidative stress, targeting of mitochondria, the | Leading to focal death of osteosarcoma cell lines |
| Bacterial-infected sepsis , Intestinal inflammation | LPS, ATP, Nigericin, Bergapten, CCCP, 3-Methyladenine | J774A.1 cells, BMDMs | Bergapten、 E.coli、C. rodentium | C57BL/6 mice | Furocoumarin phytohormone | Bergapten |  | Promoting mitochondrial autophagy and improving mitochondrial homeostasis | Attenuation of inflammatory immune responses and inhibition of NLRP3 inflammasome activation, attenuation of bacterial infections |
| Osteoarthritis | LPS, TRPV4-siRNA, Mitotempo, N-Acetylcysteine, GSK1016790A, Mdivi-1, CSA, HXK2VBD, Selective inhibitor of Ca2+/calmodulin-dependent kinase II (KN93) | Chondrocytes | Anterior cruciate ligament transection (ACLT), HC067047, Mdivi-1 | C57BL/6 mice | TRPV4 inhibition | A potent and selective TRPV4 antagonist (HC-067047) | TRPV4-CaMKII-DRP1-HK2 axis | Attenuating mitochondrial dysfunction and mtROS accumulation Reduced mitochondrial translocation and excessive mitochondrial fragmentation in DRP1 | Effectively inhibited mouse chondrocyte pyroptosis |
| Osteoarthritis | LPS, M-CSF, Opti-MEM, Absent in melanoma 2 (Aim2) , IL-3 Nigericin, ATP, MSU, Aluminum salts, Rotenone,  Lipofectamine 2000, Ploy (dA:dT), Salmonella typhimurium, Gentamycin,   Selective Pim-1 inhibitor (SMI-4a), PIM kinase inhibitor (AZD1208), NaCl, KCl, NaGluconate, KGluconate, Conditioned medium , Co-culture | BMDMs、Peritoneal macrophages, THP-1, Peripheral blood mononuclear cells | DMM, SMI-4a, PBS, Isolate cells | C57BL/6 mice | Specific inhibitor (PIM-1 inhibition) | SMI-4a |  | Attenuated mtROS accumulation in macrophages | Inhibited the mtROS/CLIC signaling pathway in macrophages, blocked ASC oligomerization and NLRP3 inflammatory vesicle activation, inhibited focal death, and exerting a protective effect on chondrocytes in a co-culture system |
| Rheumatoid arthritis | Anti-CD3/CD28 beads, MRE11A-siRNA, Mirin, MRE11A plasmid, MitoTEMPO, mtDNA, ox-mtDNA, Ref-siRNA、Aim2-siRNA, NLRP3-siRNA | CD4^＋^T cells | Synovitis Induction in Chimeric Mice、MRE11A-siRNA、Mirin、VX765 | Cg-PrkdcscidIl2rgtm1Wjl/SzJ mice | The DNA repair nuclease | MRE11A |  | reducing mitochondrial respiration and ATP production and protecting mtDNA from oxidative damage and leakage into the cytoplasm | Inhibition of caspase-1-induced cellular pyroptosis and tissue inflammation |
| Gouty arthritis | M-CSF, LPS, MSU, Resveratrol , 3-Methyladenine | BMDMs | MSU, Colchicine, Brewer, Resveratrol,PBS, Isolate BMDM cells | C57BL/6J mice | a stilbene compound widely existing  in natural plants | Resveratrol | Triggers the Pink1/Parkin path | increase the level of mitochondrial membrane potential, promote mitochondrial autophagy | Inhibition of NLRP3 inflammatory vesicle activation, inhibition of pyroptosis |
| Inflammatory diseases | M-CSF, LPS, Scoparone, ATP, Nigericin, Alum, Poly (I:C) | J774A.1 cells(Mice mononuclear macrophages), BMDMs, THP-1 cells | C. Rodentiu, E. coli, Scoparone | C57BL/6J mice | Alkaloids from the Chinese herb | Scoparone |  | Promoting mitochondrial autophagy, which removes damaged mitochondria and scavenges mtROS | Inhibition of NLRP3 inflammatory vesicle activation, attenuation of macrophage Pyroptosis, and alleviation of associated inflammatory responses |
| Inflammatory diseases | 2-mercaptoethanol, PMA, Macrophagecolony stimulating factor,  LPS, Flagellin, Poly(dA:dT), Nigericin, ATP, MSU, ACA, Fagellin | BMMs, THP-1 cells |  | C57BL/6 mice | natural compound | ACA |  | Inhibition of mtROS production and ox-mtDNA generation, the | Preventing the activation of NLRP3 inflammatory vesicles in vivo and preventing associated inflammation |
| Cardiovascular diseases cardiovascular disease | LPS, M-CSF, Nigericin, Hypoxia, Indirect co-culture | BMDMs, ;H9c2(Rat cardiomyocytes) | Isolate BMDMs | C57BL/6 mice, SLP-2-/- mice | Mitochondrial inner membrane protein | SLP-2 |  | Protection of mitochondrial function | Reducing macrophage pyroptosis and indirectly attenuating hypoxic H9c2 cell injury |

**Abbreviations:** Stomatin-like protein-2 (SLP-2); Nuclear Factor Kappa-light-chain-enhancer of Activated B cells (NF-κB ); Mouse dental pulp cell clone 6T (mDPC6T); Oxidized mitochondrial DNA (ox-mtDNA); NLRP3 inflammasome specific inhibitor (MCC950); Cyclic GMP-AMP Synthase (cGAS); OverExpression (OE); cGAS inhibitor (RU.521); Oligodeoxynucleotide F (ODN-F); Hydroxychloroquine (HCQ); Elamipretide (EPT); Elamipretide (SS-31); Clustered Regularly Interspaced Short Palindromic Repeats (CRISPR); CRISPR-associated protein 9 (Cas9); Bortezomib (BZ); pWPI:Lentiviral vectors; Gasdermin E (GSDME); N-terminal fragment of Gasdermin E with T6E mutation (N-GSDME-T6E); Plasmid Wistar Institute expressing Gasdermin E with D270A mutation (pWPI-GSDME-D270A ); Dichloroacetate (DCA); Poly[2-( N -oxide- N,N-diethylamino)ethyl methacrylate] (OPDEA); Mitochondria-targeting polymer micelle (OPDEA-PDCA); poly[2-(N-oxide-N,N-diethylamino)ethyl methacrylate] (OPDEA); MNNG-HOS (Human osteosarcoma cells); MG63 (Human osteosarcoma cells); K7M2(Osteoblasts of osteosarcoma in mice); Carbonyl cyanide-m-chlorophenyl-hydrazine (CCCP); Transient Receptor Potential Vanilloid 4 (TRPV4); Selective and potent agonist of TRPV4 (GSK1016790A); Mitochondrial division inhibitor 1 (Mdivi-1); Hexokinase II VDAC binding domain peptide (HXK2VBD); Dynamin-related protein 1 (Drp1); Interleukin-3(IL-3); Destabilization of the Medial Meniscus (DMM); Polydeoxyadenylic-thymidylic acid (Poly (dA:dT)); Meiotic recombination 11 homolog A (MRE11A); PTEN induced putative kinase 1(PINK1); Polyinosinic-polycytidylic acid (Poly(I:C)); Citrobacter rodentium (C. Rodentiu); Escherichia coli (E. coli); Phorbol 12-myristate 13-acetate (PMA); Acetoxychavicol acetate (ACA); Stomatin-like protein-2 (SLP-2)
