# Supplementary material for: Mitochondria: a key regulator of programmed cell death in OP
Source: Front Endocrinol (Lausanne). 2025 Jul 2;16:1576597. doi: 10.3389/fendo.2025.1576597 (PMC12263366; doi:10.3389/fendo.2025.1576597)
Supplement: Supplementary file 7 [file DataSheet7.docx]

**Tab.4-1 Mitochondria-Mediated Ferroptosis in the Development of Osteoporosis**

| **Diseases** | **Cells processing** | **The cells used** | **Animals handling** | **Animals used** | **Effects on mitochondria** | **Effects on bone/bone-associated cells** |
| --- | --- | --- | --- | --- | --- | --- |
| Iron overload-induced osteoporosis | Ferric ammonium citrate , Deferoxamine, Ferrostatin-1 | MC3T3-E1 | Iron dextran , Ferrostatin-1, Deferoxamine, | C57/BL6 mice | Affecting ultrastructural changes in mitochondria | Promotion of iron death in osteoblasts in vitro, inhibition of osteoblast differentiation and mineralization in vitro, and induction of osteoporosis in vivo |
| Type 2 diabetic osteoporosis | High glucose, Vitamin K2, Knockdown of SIRT1 | BMSCs | Treptozotocin solution,   High-fat and High-sugar diet. | C57/BL6 mice | Affecting mitochondrial reactive oxygen species levels in vitro | Inhibition of BMSCs iron death, restoration of bone mass and enhanced expression of iron death markers and osteogenic markers in the distal femur |
| Type 2 Diabetic Osteoporosis | High glucose, LV-FtMt-RNAi, LV-FtMt, Ferrostatin-1 | hFOB 1.19 | Deferoxamine , High-fat feed、 Streptozotocin | Sprague Dawley rats | Affecting mitochondrial autophagy | Influencing oxidative stress induced by excess ferrous ions to modulate the onset of iron death in osteoblasts |
| Iron overload induced dysfunction of BMSCs | Ferric ammonium citrate, icariin | BMSCs |  |  | Regulating both mitochondrial fusion and fission | Restore the proliferation and osteogenic differentiation of BMSCs |

**Abbreviations:** Silent Information Regulator 1(SIRT1).
