# Supplementary material for: Mitochondria: a key regulator of programmed cell death in OP
Source: Front Endocrinol (Lausanne). 2025 Jul 2;16:1576597. doi: 10.3389/fendo.2025.1576597 (PMC12263366; doi:10.3389/fendo.2025.1576597)
Supplement: Supplementary file 8 [file DataSheet8.docx]

**Tab.4-2 Targeting Mitochondria to Inhibit Ferroptosis: A Therapeutic Strategy for Bone-Related Diseases**

| **Diseases** | **Cells processing** | **The cells used** | **Animals handling** | **Animals used** | **Type of drugs** | **Drugs** | **Improving the mitochondrial pathway** | **Effects on mitochondria** | **Effects on bone/bone-associated cells** |
| --- | --- | --- | --- | --- | --- | --- | --- | --- | --- |
| Osteoporosis | Ferric ammonium citrate,  Deferoxamine,  Ferrostatin-1 | MC3T3-E1 | Iron dextran , Ferrostatin-1, Deferoxamine | C57/BL6 mice | Inhibitors of ferroptosis | Deferoxamine, Ferrostatin-1 |  | Affecting ultrastructural changes in mitochondria | Promoting osteoblastogenesis, inhibiting cellular iron death to maintain iron homeostasis, and improving osteoporosis |
| Type 2 diabetic osteoporosis | Erastin, Deferoxamine,  Ferrostatin-1, Autophagy inhibitor 3-methyladenine, Z-VAD-FMK, Necrostain-1, Luzindole, Melatonin, Nrf2-siRNA | MC3T3-E1 | Intralipids, Streptozotocin | Sprague-Dawley mouse | Hormone, Endogenous antioxidant | Melatonin | Nrf2/HO-1 | Restoring the morphology of mitochondria | Increased osteoclastogenic capacity of osteoblasts. inhibited iron death and improved bone microstructure in diabetic rats |
| Type 2 diabetic osteoporosis | High glucose , Carbonyl cyanide-m-chlorophenyl-hydrazine , CCCP, Ferrostatin-1,  LV-FtMt , LV-FtMt-RNAi, N-Acetylcysteine | hFOB1.19 | Streptozotocin, High-fat feed, Deferoxamine | Sprague-Dawley mouse | Mitochondrial ferritin | Mitochondrial ferritin | ROS/PINK1/Parkin | Inhibition of mitochondrial autophagy, thereby stabilizing iron ions in mitochondria Inhibition of oxidative stress | Reduction of oxidative stress induced by excess ferrous ions to inhibit osteoblastic iron death |
| Iron overload-induced osteoporosis | Ferric ammonium citrate , Deferoxamine,, Sodium citrate, Ascorbic acid, β-glycerophosphate | BMSCs、mouse stromal cell line ST2 | Isolate BMSCs cells | C57BL/6 mice | Reductant, ferroptosis inhibitor,  Wnt signaling agonist | Melatonin, Ferrostain-1, CHIR-99021 | Wnt signaling | Restoration of mitochondrial morphology | Specific attenuation of iron death and restoration of osteoblast differentiation |
| Osteoporosis | Macrophage colony-stimulating factor, Receptor activator for nuclear factor-κB ligand | BMMs | Lactobacillus salivarius ( Lactobacillus salivarius LI01) ,  Normal saline, Ovariectomy | BALB/c mice | Probiotic strains | Lactobacillus salivarius LI01 | CREB signaling | Affecting mitochondrial biogenesis | Enhancement of GSH synthesis from scratch and alleviation of osteoporosis in mice |
| Wound healing | Glutathione Peroxidase 4 Inhibitor (RSL3), δ-Tocotrienol | BMSCs, NIH-3T3, PAM-212, C166 | δ-TT-BMSCs, BMSCs | BALB/c mice | Cells after pretreatment | δ-TT-BMSCs | Down-regulating the expression of BACH1 and activating PI3K/AKT signaling | Reversed the decrease in mitochondrial membrane potential, lowered the level of ROS, and altered the mitochondrial microstructure, the | Increase cell viability, inhibit cellular iron death, promote wound healing, and |
| Steroid-induced osteonecrosis of the  femoral head | Methylprednisolone, Melatonin, N-Acetylcysteine, Vitamin C, Luzindole, shGDF15 | BMSCs | Glucocorticoid, Melatonin , N-acetyl-5-methoxytryptamine,  Methylprednisolone , Sodium succinate, Lipopolysaccharide ,  Pentobarbital sodium | Sprague-Dawley mouse | Endocrine hormone | Melatonin | Through GDF15-mediated signaling | Restoring the mitochondrial membrane potential and reducing the level of ROS | Inhibited iron death in BMSCs and restored the osteogenic differentiation ability of the cells, thereby effectively reducing Methylprednisolone-induced Steroid-induced osteonecrosis of the  femoral head in rats |
| Chemical damage | Methylglyoxal, Actein, Aminoguanidine, β-glycerophosphate, Ascorbic acid | MC3T3-E1 |  |  | Plant extracts | Actein | By increasing the levels of PGC-1α, NRF-1, and Nitric oxide | Enhancement of mitochondrial biogenesis factors and reduction of MG-induced mitochondrial dysfunction in osteoblast MC3T3-E1 | Reduction of mitochondrial reactive oxygen species production in cells may be one of the mechanisms by which Actein enhances the anti-inflammatory and antioxidant bioactivities of osteoblasts and reduces cellular damage |
| Age-related bone loss | Antimycin A, Sciadopitysin | MC3T3-E1 |  |  | Plant extracts | Sciadopitysin |  | Protecting mitochondria against a burst of oxidative stress | Reduce or prevent osteoblasts degeneration |

**Abbreviations:** Mitochondrial ferritin (FtMt); Reactive oxygen species (ROS); Wingless-Type MMTV Integration Site Family (Wnt); cAMP response element‐binding protein (CREB); BTB and CNC homology 1 (BACH1); Growth differentiation factor 15 (GDF15); Short hairpin RNA targeting Growth differentiation factor 15 ( shGDF15 ); Nuclear respiratory factor 1 (NRF-1); Peroxisome proliferator-activated receptor gamma coactivator 1 alpha (PGC-1α); NIH Swiss Mouse Embryonic Fibroblast Cell Line 3T3 (NIH-3T3); Murine Epidermal Keratinocyte Cell Line PAM-212 (PAM-212); Mouse Embryonic Yolk Sac Endothelial Cell Line C166 (C166).
