# Supplementary material for: Mitochondria: a key regulator of programmed cell death in OP
Source: Front Endocrinol (Lausanne). 2025 Jul 2;16:1576597. doi: 10.3389/fendo.2025.1576597 (PMC12263366; doi:10.3389/fendo.2025.1576597)
Supplement: Supplementary file 9 [file DataSheet9.docx]

**Tab.5-1 Mitochondria and Selective Autophagy in the Pathogenesis of OP**

| **Diseases** | **Cells processing** | **The cells used** | **Animals handling** | **Animals used** | **Effects on mitochondria** | **Effects on bone/bone-associated cells** |
| --- | --- | --- | --- | --- | --- | --- |
| Osteoporosis | RANKL, M-CSF, Isobutylmethylxanthine , Dexamethasone, Indomethacin and insulin,  Ascorbate-2,β-glycerol phosphat, Dexamethasone； LV^sh-LRRc17^、LV^ov-LRRc17^、LV-GFP H_2_O_2_. | BMSC,s BMMs, MC3T3-E1 | Isolate BMSCs cells | C57BL/6 mice | Modulates mitochondrial dysfunction, Attenuates mitochondrial damage, Influences mitochondrial autophagy, and | Protecting BMSCs from the effects of aging and alleviating bone loss in OVX mice |
| Osteoporosis | Mouse serum albumin, Advanced oxidation protein products, Apocynin, Rapamycin, MitoTEMPO, 3-Methyladenine, Bafilomycin A1, shPINK1, Scrambled shRNA | MC3T3-E1 | Advanced oxidation protein products, Mouse serum albumin, Rapamycin, PBS | Mice | Stimulate mitophagy to eliminate ROS and damaged mitochondria | Inhibit osteoblast apoptosis, thereby ameliorating bone loss, deterioration of bone microstructure, and loss of bone mineral density |
|  | Ferutinin, Knockdown of Klf2, Knockdown of Atg7, Knockdown of beclin1, H_2_O_2_, _,_ | DPSCs |  |  | Induce mitophagy and impair mitochondrial metabolic function | Promotes osteogenic differentiation of cells |
| Osteoporosis | Ascorbic acidd, β-glycerophosphate , Knockdown of PINK1 | MC3T3-E1 | Ovariectomy, Knockout of PINK1 | C57BL/6 mice | PINK1 knockout disrupts mitochondrial homeostasis, impairs mitochondrial ROS production, and compromises mitochondrial calcium handling. | PINK1 knockout mice exhibit decreased bone mass and impaired collagen synthesis. Furthermore, PINK1 downregulation inhibits osteoblast differentiation. |
| Periodontitis | M-CSF, RANKL, Spermidine, N-acetyl-l-cysteine, | BMMs | Ligature, | C57BL/6 mice ,PINK1 KO mice | PINK1 deficiency leads to mitochondrial dysfunction, accompanied by impaired mitochondrial respiration and elevated intracellular Ca^2+^ and lactate levels. | PINK1 knockout increases osteoclasts in mouse periodontal tissue. PINK1 silencing or deletion enhances osteoclast differentiation and bone resorption in vitro |
| Osteoporosis | M-CSF , RANKL, E_2,_, Sirt3 inhibitor (LC-0296) | BMMs | Ovariectom, DMSO/PBS mixture, The Sirt3 inhibitor (LC-0296), | B6/Sv129 mice, C57BL/6 mice, ERα^ΔLysM^ mice, Sirt3 KO mice | The deficiency of Sirt3 affects mitochondrial function, leading to a reduction in oxidative phosphorylation and mitophagy | The absence of Sirt3 leads to osteoclast differentiation and resorption activity, thereby affecting bone absorption |
| Bone defects | Penicillin, Streptomycin, ascorbic acid, β-glycerophosphate, Dexamethasone, MBGNs , Cu-MBGNs, Conditioned medium of MBGNs; Conditioned medium of Cu-MBGNs, Bafilomycin A1 , Cyclosporin A, N-acetylcysteine, Mdivi-1 | BMSCs | Cu/L-ascorbic acid complex, MBGNs, Cu-MBGNs, Femoral defect, PBS | C57BL/6J mice, (Prx1-Cr; Atg5^f/f^ )mice | Cu-MBGNs promoted mitochondrion fission via activating dynamin related protein 1 to reinforce mitophagy pathway | Cu-MBGNs accelerates the process of bone regeneration |
| Diabetic osteoporosis | β-glycerophosphate disodium salt hydrate, L-ascorbic acid , Low glucose , High glucose, BMP9, siRNA, PINK1 siRNA, DRP1 siRNA, Mitochondria-targeted mKeima-Red expression plasmid , PINK1 expression plasmid | MC3T3-E1 | Streptozotocin, BMP9 | C57BL/6 mice | The upregulation of PINK1/DRP1 pathway activated mitophagy | Activation of mitophagy through the PINK1/DRP1 pathway can restore the differentiation capacity of osteoblasts, improving bone quality and bone mineral density |
|  | Overexpression of MFN2 T111E-S442E, Overexpression of MFN2 T111A-S442A | BMMs |  | (LysMc/c; Mfn2+/+; cre-only) mice, (LysMc/c; Mfn2fl/fl; Mfn2 cKO) mice,  cre-only (LysM^c/c^;(Mfn1/2)^+/+^) mice, (LysM^c/c^; (Mfn1/2)^fl/+^ = ctrl) mice, (LysM^c/c^; (Mfn1/2)^fl/fl^; dcKO)mice | MFN2 mediates the tethering between mitochondria and the endoplasmic reticulum (ER), thereby promoting mitophagy | Regulation of osteoclast differentiation |
| Osteoporosis | H_2_O_2_, [Apelin](https://www.sciencedirect.com/topics/biochemistry-genetics-and-molecular-biology/apelin)-13, CsA, A[scorbic acid](https://www.sciencedirect.com/topics/pharmacology-toxicology-and-pharmaceutical-science/ascorbic-acid) , β-glycerophosphate, 3‐isobutyl‐1‐methylxanthine, Insulin, Dexamethasone, Knockdown of AMPK-α | BMSCs | Isolate BMSCs cells, Ovariectomy, Apelin-13 | Sprague-Dawley rat | Mitophagy is activated | Ameliorates oxidative stress, enhances osteogenic function, and restores bone mass and microarchitecture in ovariectomy rats. |
| Disease | Cell processing | The cells used | Animal handling | Animals used | Effects on mitochondria | Effects on bone/bone-associated cells |
| Osteoporosis | RANKL, M-CSF, Isobutylmethylxanthine , Dexamethasone, Indomethacin and insulin,  Ascorbate-2,β-glycerol phosphat, Dexamethasone； LV^sh-LRRc17^、LV^ov-LRRc17^、LV-GFP H_2_O_2_. | BMSC,s BMMs, MC3T3-E1 | Isolate BMSCs cells | C57BL/6 mice | Modulates mitochondrial dysfunction, Attenuates mitochondrial damage, Influences mitochondrial autophagy, and | Protecting BMSCs from the effects of aging and alleviating bone loss in OVX mice |
| Osteoporosis | Mouse serum albumin, Advanced oxidation protein products, Apocynin, Rapamycin, MitoTEMPO, 3-Methyladenine, Bafilomycin A1, shPINK1, Scrambled shRNA | MC3T3-E1 | Advanced oxidation protein products, Mouse serum albumin, Rapamycin, PBS | Mice | Stimulate mitophagy to eliminate ROS and damaged mitochondria | Inhibit osteoblast apoptosis, thereby ameliorating bone loss, deterioration of bone microstructure, and loss of bone mineral density |
|  | Ferutinin, Knockdown of Klf2, Knockdown of Atg7, Knockdown of beclin1, H_2_O_2_, _,_ | DPSCs |  |  | Induce mitophagy and impair mitochondrial metabolic function | Promotes osteogenic differentiation of cells |
| Osteoporosis | Ascorbic acidd, β-glycerophosphate , Knockdown of PINK1 | MC3T3-E1 | Ovariectomy, Knockout of PINK1 | C57BL/6 mice | PINK1 knockout disrupts mitochondrial homeostasis, impairs mitochondrial ROS production, and compromises mitochondrial calcium handling. | PINK1 knockout mice exhibit decreased bone mass and impaired collagen synthesis. Furthermore, PINK1 downregulation inhibits osteoblast differentiation. |
| Periodontitis | M-CSF, RANKL, Spermidine, N-acetyl-l-cysteine, | BMMs | Ligature, | C57BL/6 mice ,PINK1 KO mice | PINK1 deficiency leads to mitochondrial dysfunction, accompanied by impaired mitochondrial respiration and elevated intracellular Ca^2+^ and lactate levels. | PINK1 knockout increases osteoclasts in mouse periodontal tissue. PINK1 silencing or deletion enhances osteoclast differentiation and bone resorption in vitro |
| Osteoporosis | M-CSF , RANKL, E_2,_, Sirt3 inhibitor (LC-0296) | BMMs | Ovariectom, DMSO/PBS mixture, The Sirt3 inhibitor (LC-0296), | B6/Sv129 mice, C57BL/6 mice, ERα^ΔLysM^ mice, Sirt3 KO mice | The deficiency of Sirt3 affects mitochondrial function, leading to a reduction in oxidative phosphorylation and mitophagy | The absence of Sirt3 leads to osteoclast differentiation and resorption activity, thereby affecting bone absorption |
| Bone defects | Penicillin, Streptomycin, ascorbic acid, β-glycerophosphate, Dexamethasone, MBGNs , Cu-MBGNs, Conditioned medium of MBGNs; Conditioned medium of Cu-MBGNs, Bafilomycin A1 , Cyclosporin A, N-acetylcysteine, Mdivi-1 | BMSCs | Cu/L-ascorbic acid complex, MBGNs, Cu-MBGNs, Femoral defect, PBS | C57BL/6J mice, (Prx1-Cr; Atg5^f/f^ )mice | Cu-MBGNs promoted mitochondrion fission via activating dynamin related protein 1 to reinforce mitophagy pathway | Cu-MBGNs accelerates the process of bone regeneration |
| Diabetic osteoporosis | β-glycerophosphate disodium salt hydrate, L-ascorbic acid , Low glucose , High glucose, BMP9, siRNA, PINK1 siRNA, DRP1 siRNA, Mitochondria-targeted mKeima-Red expression plasmid , PINK1 expression plasmid | MC3T3-E1 | Streptozotocin, BMP9 | C57BL/6 mice | The upregulation of PINK1/DRP1 pathway activated mitophagy | Activation of mitophagy through the PINK1/DRP1 pathway can restore the differentiation capacity of osteoblasts, improving bone quality and bone mineral density |
|  | Overexpression of MFN2 T111E-S442E, Overexpression of MFN2 T111A-S442A | BMMs |  | (LysMc/c; Mfn2+/+; cre-only) mice, (LysMc/c; Mfn2fl/fl; Mfn2 cKO) mice,  cre-only (LysM^c/c^;(Mfn1/2)^+/+^) mice, (LysM^c/c^; (Mfn1/2)^fl/+^ = ctrl) mice, (LysM^c/c^; (Mfn1/2)^fl/fl^; dcKO)mice | MFN2 mediates the tethering between mitochondria and the endoplasmic reticulum (ER), thereby promoting mitophagy | Regulation of osteoclast differentiation |
| Osteoporosis | H_2_O_2_, [Apelin](https://www.sciencedirect.com/topics/biochemistry-genetics-and-molecular-biology/apelin)-13, CsA, A[scorbic acid](https://www.sciencedirect.com/topics/pharmacology-toxicology-and-pharmaceutical-science/ascorbic-acid) , β-glycerophosphate, 3‐isobutyl‐1‐methylxanthine, Insulin, Dexamethasone, Knockdown of AMPK-α | BMSCs | Isolate BMSCs cells, Ovariectomy, Apelin-13 | Sprague-Dawley rat | Mitophagy is activated | Ameliorates oxidative stress, enhances osteogenic function, and restores bone mass and microarchitecture in ovariectomy rats. |

**Abbreviations:** leucine-rich repeat containing 17 (LRRc17); Dental pulp stem cells (DPSCs); Kruppel-like factor 2 (KLF2); Autophagy Related 7 (ATG7); Sirtuin-3 (Sirt3); Mesoporous bioactive glass nanoparticles (MBGNs) ; Cu-doped mesoporous bioactive glass nanoparticles (Cu-MBGNs); Bone morphogenetic protein 9 (BMP9); Dynamin-related protein 1 (DRP1)
