# Supplementary material for: Mitochondria: a key regulator of programmed cell death in OP
Source: Front Endocrinol (Lausanne). 2025 Jul 2;16:1576597. doi: 10.3389/fendo.2025.1576597 (PMC12263366; doi:10.3389/fendo.2025.1576597)
Supplement: Supplementary file 10 [file DataSheet10.docx]

**Tab.5-2 Targeting Mitophagy: A Therapeutic Strategy for Bone-Related Disorders**

| **Diseases** | **Cells processing** | **The cells used** | **Animals handling** | **Animals used** | **Type of drugs** | **Drugs** | **Improving the mitochondrial pathway** | **Effects on mitochondria** | **Effects on bone/bone-associated cells** |
| --- | --- | --- | --- | --- | --- | --- | --- | --- | --- |
| Osteoporosis | PINK1-siRNA, PINK1-pcDNA3 | MC3T3-E1 | Ovariectomy、 | C57BL/6mice, Pink1 KO mice | A serine/threonine-protein kinase  in mitochondria | PINK1 | PINK1 | May alter the levels of ROS in mitochondria by affecting the quality of mitochondrial function and mitochondrial autophagy | Affecting osteogenic differentiation of osteoblasts |
| HIV-induced osteoporosis | Tat , Nef , N-acetyl cysteine, Parthenolide, Rapamycin | BMSCs |  |  | Antioxidant treatment, Autophagy agonists | Rapamycin, N-acetyl cysteine | Antioxidant, Activates autophagy | Affecting levels of oxidative stress and mitochondrial dysfunction, altering levels of autophagy | Affecting levels of proliferation, oxidative stress and senescence in MSCs |
| Osteoporosis | 17β-estradiol, Glutamine, bFGF, Selective GPR30 antagonist (G25), Mitogen-activated protein (MAP) kinase (ERK1/2) inhibitor(U0126,), | MC3T3-E1 |  |  | Hormone | 17β-estradiol | GPR30-ERK1/2 | Accumulation of mitochondrial autophagosomes, enhancement of mitochondrial autophagy, the | Increased MC3T3-E1 cell activity and protection of osteoblasts |
| Senile osteoporosis | Ascorbic acid, β glycerol phosphate disodium salt hydrate, miR-21-5p expression | BMSCs |  | BALB/c mice, SAM/P6 mice (senescence-accelerated mice prone 6) | microRNA | miR-21-5p |  | Reducing autophagy and modulating mitochondrial network dynamics | Improvement of viability and proliferative activity, osteogenic differentiation capacity, and inhibition of osteoclastogenesis in senile osteoporotic BMSCs, improved bone regeneration in mice with an aged phenotype |
| Imbalance of calcium and magnesium in bones | Dexamethasone, β-glycerophosphate, Ascorbic  acid-2-phosphate, 3-MA, Exogenous ATP | hBMSCs |  |  | Compound | Exogenous ATP |  | Affecting the level of mitochondria-associated autophagy | Reversing the inhibitory effect of high Mg2+ on the mineralization of hBMSCs |
| Early steroid-induced osteonecrosis of the femoral | H_2_O_2_, Lv-Parkin-EGFP, p53-shRNA | BMSCs | lipopolysaccharide 、methylprednisolone、 transplanted the BMSCs/XACB、 transplanted the XACB | New Zealand white rabbits | Cell transplantation | Parkin-shp53 BMSCs | upregulating Parkin and downregulating p53 expression | Enhanced mitochondrial autophagy and reduced the accumulation of damaged mitochondria in the cell | Effectively improving the repair of early steroid-induced femoral head necrosis by BMSCs |

**Abbreviations:** Transcription activator (TAt); Negative regulatory factor (Nef); Basic fibroblast growth factor (bFGF); G protein-coupled receptor 30 (GPR30); Extracellular-regulated kinase 1/2 (ERK1/2); Enhanced Green Fluorescent Protein (EGFP)
